# Reconstitution of GABA, Glycine and Glutamate Transporters

**DOI:** 10.1007/s11064-021-03331-z

**Published:** 2021-04-27

**Authors:** Niels Christian Danbolt, Beatriz López-Corcuera, Yun Zhou

**Affiliations:** 1 Neurotransporter Group, Department of Molecular Medicine, Institute of Basic Medical Sciences, University of Oslo, 0317 Oslo, Norway; 2 Departamento de Biología Molecular, Universidad Autónoma de Madrid, Madrid, Spain; 3 Centro de Biología Molecular “Severo Ochoa” Consejo Superior de Investigaciones Científicas, Universidad Autónoma de Madrid, Madrid, Spain; 4 IdiPAZ, Hospital Universitario La Paz, Madrid, Spain

**Keywords:** GABA transporter, Glutamate transporter, Glycine transporter, Reconstitution, Liposomes, Scientific history

## Abstract

In contrast to water soluble enzymes which can be purified and studied while in solution, studies of solute carrier (transporter) proteins require both that the protein of interest is situated in a phospholipid membrane and that this membrane forms a closed compartment. An additional challenge to the study of transporter proteins has been that the transport depends on the transmembrane electrochemical gradients. Baruch I. Kanner understood this early on and first developed techniques for studying plasma membrane vesicles. This advanced the field in that the experimenter could control the electrochemical gradients. Kanner, however, did not stop there, but started to solubilize the membranes so that the transporter proteins were taken out of their natural environment. In order to study them, Kanner then had to find a way to reconstitute them (reinsert them into phospholipid membranes). The scope of the present review is both to describe the reconstitution method in full detail as that has never been done, and also to reveal the scientific impact that this method has had. Kanner’s later work is not reviewed here although that also deserves a review because it too has had a huge impact.

## Introduction

The mammalian genome contains more than 400 genes encoding various solute carrier (SLC) transporter proteins. Together, these proteins are responsible for most of the transport of small hydrophilic molecules across biological membranes. Thus, these proteins are essential for absorption of nutrients from the intestine, for reabsorption of filtered compounds in the kidneys, waste removal, ion transport and even neurotransmitter inactivation [1–3].

Although detailed information was lacking, it was obvious from the very beginning of modern biochemical research that amino acid transport across biological membranes had to be important. The challenge was to find ways to study these transport processes. In contrast to water soluble enzymes, which could be purified and studied in isolation from other cellular components and cellular processes, studies of transporters were limited to the use of intact organisms (eukaryotic cells, yeast or bacteria), tissue slices containing intact cells or fresh homogenates containing resealed cellular fragments (e.g. synaptosomes representing pinched off nerve terminals). This hampered progress as the properties of the transport processes themselves were hard to distinguish from indirect effects due to changes of energy metabolism and the activity of ion channels affecting ion gradients and membrane potentials.

**Fig. 1:**
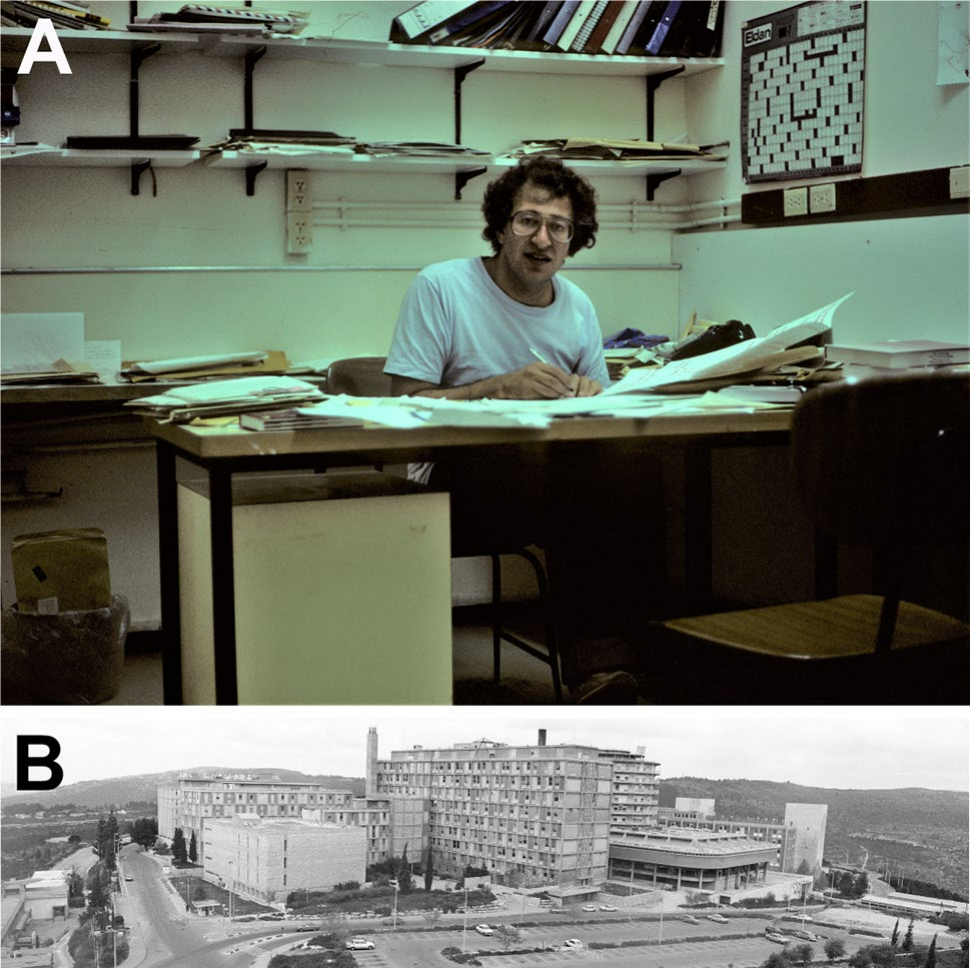
**a** Baruch Kanner in his office in 1987 at **b** the Hadassah Medical School, the Hebrew University, Ein Kerem, Jerusalem, Israel. Copyright: Neurotransporter.org; reproduced with permission

**Fig. 2:**
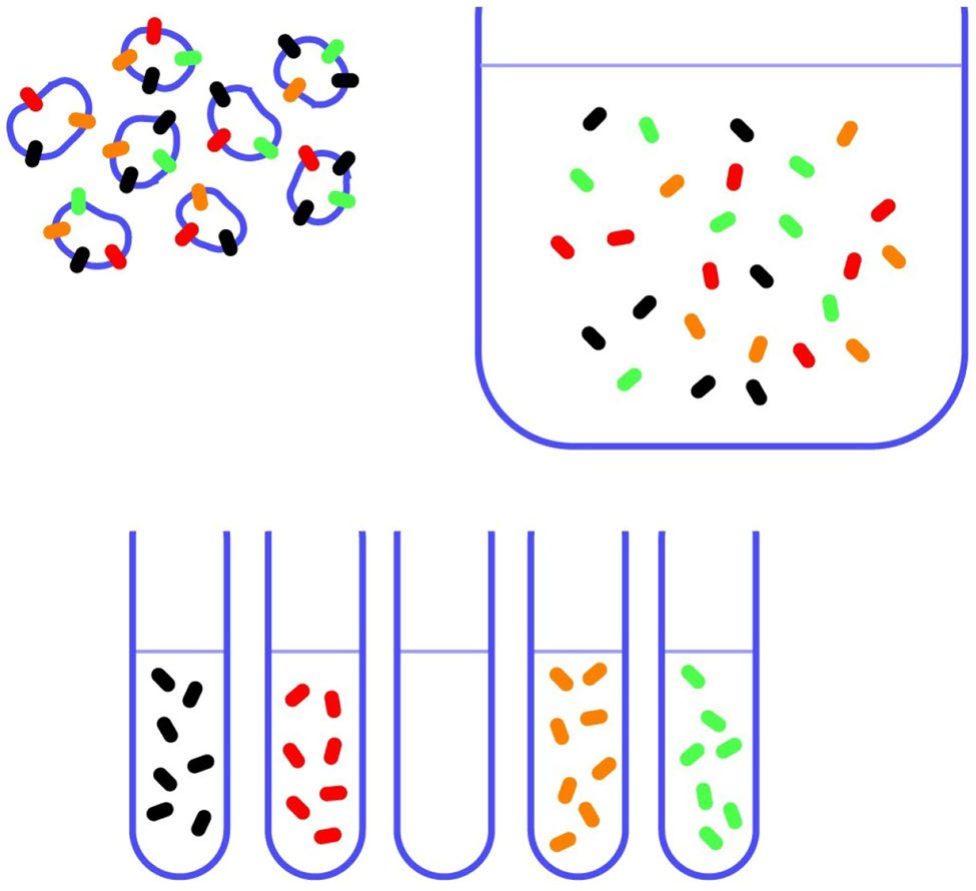
A schematic illustration of the purpose of solubilization and the need for a method to detect the transporter of interest after separation. Copyright: Neurotransporter.org; reproduced with permission

**Fig. 3:**
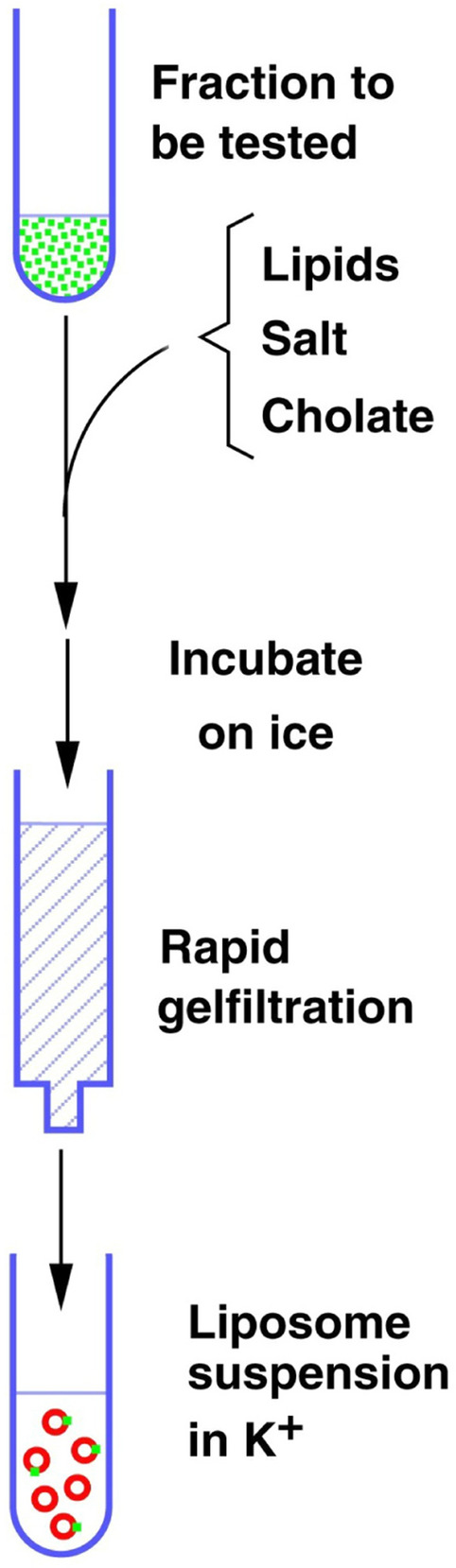
Schematic illustration of the reconstitution process. Solubilized transporter proteins are mixed with the reconstitution mixture containing lipids, salt and cholate. After incubation on ice, cholate is removed by gel filtration and the liposomes form spontaneously. The fluid in which they are suspended becomes trapped inside and therefore become the internal medium. Copyright: Neurotransporter.org; reproduced with permission

Kanner (Fig. 1) realized that new methods were required for membrane transport research. He began to make membrane vesicles which he loaded with ions. Thus, he developed procedures that were independent of the cellular ATP production. Although important data were obtained from this (e.g. [4–8]), the transporters could still not be studied in full isolation from other membrane proteins; e.g. ion channels which made the plasma membrane vesicles leaky. Further, the studies were limited to those membrane fragments that could spontaneously reseal to form closed compartments (for example importance, see Fig. 7 and for discussion see: [9]). In the late 1970s Kanner began to solubilize the plasma membranes (Fig. 2). This implied dissolving the lipid membranes so that that the various membrane proteins were no longer kept together by the lipids. However, as transport function has no meaning without closed compartments, it was necessary to develop a method to reconstitute the transporters in new (artificial) cell membranes (liposomes). Kanner quickly succeeded in reconstituting both GABA and glutamate transport activities from rat brain [8, 10, 11] as he was experienced with methods for membrane protein reconstitution from his studies in Efraam Racker’s laboratory that involved studies of bacterial proteins (e.g. [12]). This was a major advance. Firstly, the membrane proteins could be diluted in excess lipids so that liposomes harboring a transporter of interest might not contain (or at least have a considerably reduced amounts of) interfering membrane proteins such as ion channels. Secondly, because the solubilized proteins were free in solution, it was now possible for the first time to attempt purification of the transporter proteins (Fig. 2) as (a) they could now be separated from other proteins, and (b) the various protein fractions could be assessed for transporter protein content by reconstituting the transporters into artificial membranes (liposomes; Fig. 3).

The scope of the present review is firstly to describe the reconstitution procedure is sufficient detail to allow it to be reproduced in other laboratories as that has never been done, and, secondly, to review studies in which the method has been used to illustrate its impact.

## Overview

The reconstitution method described here is a modification of a published procedure [13] which in turn was based on earlier work [11, 14, 15]. The method is suitable for reconstitution of glutamate transporters [13, 16–19], GABA transporters [14, 20], glycine transporters [21] and the serotonin transporter [22] although, as pointed out below, the GABA and glycine transporters have somewhat different requirements than the glutamate transporters.

The liposomes obtained with this procedure are mainly unilamellar and have a somewhat variable diameter (100–300 nm) [23]. Thus, they are larger than synaptic vesicles (40 nm in diameter), but much smaller than synaptosomes (0.4–1 μm). For comparison, a human red blood cell is about 7 μm in diameter.

The liposomes can be prepared from frozen brain membranes or tissue, but once made, the liposomes do not tolerate freezing and should be stored on ice until used. Freezing leads to formation of ice crystals which may render the membrane leaky (for a description of freezing of biological systems see: [24]). Being tight compartments, the liposomes are also osmotically sensitive. Exposed to air and stored on ice, glutamate transport activity is virtually unchanged the first 24 h and may be stable up to 48 h. If the liposomes are stored on ice protected from air, high glutamate transport activity has been recorded after 5 days (D. Trotti and N.C. Danbolt, unpublished).

**Fig. 4:**
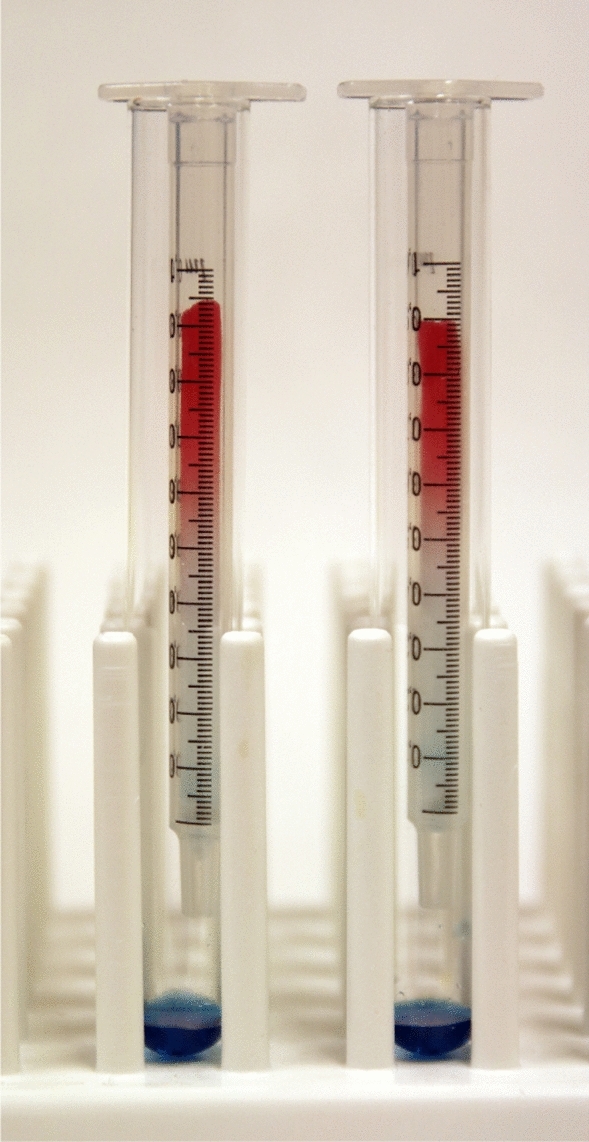
Illustrating the principle of gel filtration in removing detergents during reconstitution. A mixture (200 µl) of a low molecular mass dye (phenol red) and a high molecular mass compound (blue-dextran 2000) were added to 1 ml spin columns which are packed with Sephadex G-50 in internal medium. Copyright: Neurotransporter.org; reproduced with permission

The liposomes are formed when the detergent of choice (cholate) is removed by gel filtration (Figs. 3 and 4). This gives complete control of the internal medium as the liposomes will enclose the gel filtration buffer and also be suspended in the same. Further, the buffer on the outside of the liposomes can be removed by another gel filtration. Thus, it is also possible to control the external medium. While electrophysiological techniques detect transporter induced electrical currents, the liposomes can be used to study electroneutral substrate exchange; uptake of external substrate and release of internal substrate [16, 19, 25].

Heteroexchange studied this way can also be used to test if an unlabeled novel compound is actually translocated because internal accumulation of a labeled substrate added to external medium will imply that the two compounds have been exchanged with each other [23].

The main limitations of the assay are the temporal resolution (time needed for starting and stopping the uptake reaction) and the fact that the driving forces (the electrochemical gradients) are not renewed because there is no Na^+^K^+^-ATPase activity. This means that the transport is not fully linear with time (see Fig. 14).

## Detailed Description of the Method

### Purification of Cholic Acid by Recrystallization

The cholic acid (CAS 81-25-4) used for the purification and reconstitution of the above mentioned transporters has been relative cheap cholic acid from bovine bile (e.g. Sigma C-1129) which gives a yellow solution due to impurities. Therefore, the cholic acid was purified before use. This was done by recrystallization as described [26]: One kilogram of cholic acid was dissolved in six liters of 70% (v/v) ethanol by heating. Activated charcoal (about 50−25 mg/ml) was added when all the cholic acid was dissolved. Stirring was continued and the solution was boiled for about 30 min (*NB: Fire hazard as ethanol is flammable!*) and then quickly filtered through a double layer of Whatman #1 filter-paper. The filter paper was washed with 70% (v/v) ethanol prior to use in order to remove detergents and other chemicals. The charcoal treatment was repeated until the solution was colorless. After the last filtration, the solution was allowed to cool (at 4 °C) over-night. Crystals would then form. They were likely to be grey due to charcoal. After collecting the crystals by filtration, they were dissolved in new 70% (v/v) ethanol (about 4 ml per gram wet crystals) while heating. The solution was filtered and allowed to crystalize as above. This was repeated until the crystals were colorless (usually three crystallizations) and thereby free of charcoal. The final crystals were dried until they no longer smelled of ethanol. The yield was about 50%. The final product is stable as illustrated by the fact that the batch of cholic acid purified by Danbolt December 31st, 1991, is still used in 2020.

### Preparation of 20% (w/v) Cholate Stock Solution

The cholate stock solution was made by adding purified water to 20 g of the above purified cholic acid crystals up to about 80 ml. Then 4.2 ml 10 M sodium hydroxide (NaOH) was added and the mixture was stirred for a couple of hours at room temperature. More NaOH was added until all the crystals were dissolved. Care was taken to avoid adding excess NaOH. Finally, the pH was checked to be in the range 7.2–7.4 and water was added to a final volume of f 100 ml. This solution was stored at − 20 °C. If stored at this temperature in an inert container properly closed, it can be kept for several years. NB: This solution is a solution of sodium cholate, but the concentration is 490 mM: 20% (w/v) based on cholic acid and not of sodium cholate.

### Extraction of Crude Bovine Brain Lipids

The brain lipids used for reconstitution was prepared as described [27]. Briefly, bovine brains were homogenized in 20 volumes of chloroform-methanol 2:1 (v/v). The homogenate was filtered through a filter paper (Whatman Chromatography paper no. 3) which was first defatted by washing with chloroform–methanol. The filtrate was subsequently mixed vigorously with 0.2 volumes 0.04 % (w/v) magnesium chloride (MgCl_2_). This resulted in a phase separation, and the two phases could be separated from each other either by centrifugation for 20 min or by standing in a separation funnel overnight. The upper phase was discarded. When centrifugation was used, the interphase had to be washed three times with chloroform–methanol–0.04 % MgCl_2_ (3:48:47). The washing fluid was discarded, and the lower phase was taken to dryness in an evaporator. Stock solutions were prepared by dissolving the lipids to a concentration of 100 g/l in chloroform-methanol 2:1 (v/v). The solution was stored under nitrogen in sealed brown glass bottles at − 20 °C. In this form, they are stable enough to be used for reconstitution of GABA and glutamate transporters in liposomes. The batch of lipids which was produced in Oslo by Danbolt September 10th, 1991, is still in use 30 years later! When preparing a new batch, it should be tested it in order to determine the optimal amount of brain lipids to be used in the reconstitution mixture. *Some safety considerations: Chloroform (CAS 67-66-3) is harmful, and waste should be collected. Methanol (CAS 67-56-1) is both flammable and harmful and waste should be collected. Finally, always consider the possibility that brain tissue is infected, e.g. with prions, so good laboratory practices should always be maintained *[28, 29].

### Asolectin (Phospholipid) Stock Solution

Asolectin stock solutions were prepared by dissolving 35.6 g soybean asolectin (mostly l-α-phosphatidylcholine; Sigma P-3644) in chloroform-methanol (80 + 20) to a total volume of 100 ml. Stock solutions of lipids were kept under nitrogen atmosphere in brown bottles at − 20 °C like the brain lipids above.

### The Reconstitution Mixture

The original reconstitution procedure involved several steps. The first was to prepare a lipid suspension by sonicating dried lipids in “dialysis buffer” consisting of 0.1 M potassium phosphate (KPi), 5 mM Tris-SO_4_, 10 mM MgSO_4_, 5 mM EDTA and 1 % (v/v) glycerol. This sonication had to be done in a bath sonicator (and not with a tip-sonicator), and was both somewhat tricky and time-consuming. The next was to mix the lipid solution with solubilized transporters, and adding sodium chloride (NaCl) and cholate in this order. After incubation on ice (15 min) the mixture was gel filtered to remove the cholate.

When developing the procedure to isolate glutamate transporters [13], reduction of experimental complexity became imperative as the purification process involved three chromatographic steps that had to be completed quickly due to inactivation of the transport activity. Danbolt therefore introduced the “reconstitution mixture” which contained all the required components, including cholate. This meant that three pipetting steps became one (100 µl solution with solubilized transporters + 150 µl reconstitution mixture). Further, the reconstitution mixture was faster to make because the time-consuming sonication step was eliminated. Because of the cholate, the reconstitution mixture only required whirl mixing in order to suspend the lipids. The “dialysis buffer” in which the lipids were re-suspended was also simplified in that the Tris-SO_4_ and MgSO_4_ were omitted. The glycerol was kept as that appeared to reduce inter experimental variability possibly because it is a mild antioxidant [30].

The reconstitution method is based on the cheap type of phospholipids described above, but synthetic phospholipids (unpublished) containing one polyunsaturated and one saturated fatty acid (e.g. l-α-lecithin 18:0 20:4 + 1-stearoyl-2-arachidoyl (1-acyl-2-acyl-sn-glycero-3-phosphocholine)) also work. l-α-lecithin, where both the fatty acids are saturated or both are polyunsaturated, are unsuitable (D. Trotti and N.C. Danbolt, unpublished). The addition of crude brain lipids increases the transport activity of GABA, glycine and glutamate, but is not essential for glutamate uptake. Some of the effects of the brain lipids are due to cholesterol [31]. The importance of the lipid composition is higher for purified transporters than for crude detergent extracts of brain tissue as these also contain lipids. Further, several transporters, especially EAAT2 and the glycine transporter GlyT2, are membrane proteins associated with lipid-raft microdomains and probably in addition to cholesterol they require the presence of sphingolipids in their lipid islands [32–34]. For simplicity, the description below only refers to the asolectin-brain lipids mix.

*How to make the reconstitution mixture?* Mix 480 µl of the above described asolectin stock solution with 240 µl the above described brain lipid stock solution. Then dry the lipids with a stream of nitrogen and subsequently by keeping them under high vacuum (1 h or over-night). Add 4 ml 0.1 M KPi with 1% (v/v) glycerol and then 89 µl 20% (w/v) cholate. To suspend the lipids without oxidizing them, flush the tube with nitrogen and seal it, whirl mix until all the lipid are removed from the tube walls and a homogenous milky white mixture has been obtained free of visible particles. Then, add 1 ml 3 M NaCl for mixtures intended for reconstitution of GABA and glutamate transporters, or 1 ml 5 M NaCl for reconstitution of GlyT2 [35]. Store the reconstitution under nitrogen on ice if it is to be used within 24 h of preparation. The mixture may be frozen, but the highest transport activities are obtained with fresh mixtures.

### Solubilization of Glutamate, GABA and Glycine Transport Activity

The term “solubilization” is defined as a process by which the native biological membranes are dissolved so that the proteins imbedded in them come free. To achieve efficient solubilization, the detergents must be used in concentrations above the critical micellar concentration (CMC) [36–39]. After centrifugation, the ensuing supernatant will contain mixed micelles composed of detergent molecules, lipid molecules and proteins. The minimum concentrations of 3-[(3-Cholamidopropyl)dimethylammonio]-1-propanesulfonate (CHAPS; CAS 75621-03-3 [40]) and cholate are, respectively, 20 mM and 1.2% (w/v), provided sufficient salt is present. The transporter proteins tolerate higher detergent concentrations, but the rapid gel filtration that the reconstitution procedure depends on, has limitations as explained below.

It should be added here, that after this method was developed, many different detergents have emerged. Some of which are efficient for transporter solubilization (e.g. [41]).

Extraction of brain membrane proteins for isolation of glutamate transporters [13].

- Thaw 1 aliquot of 1 ml brain membranes rapidly on 37 °C water bath.
- Then add 2.14 ml solubilization buffer (containing either 33 mM CHAPS or 2% cholate; 0.1 M sodium phosphate buffer pH 7.4, 10 mM EDTA, 0.5 mg/ml sodium azide).
- Add 35 µl 0.1 M phenylmethanesulfonyl fluoride (PMSF; CAS 329-98-6) from a freshly prepared solution in dimethyl sulfoxide (CAS 67-68-5).
- Add 344 µl saturated ammonium sulfate (to 10% saturation) or 3 M NaCl per ml membranes.
- Total volume should be 3.48 ml (per ml original membranes) and the final protein concentration will be about 10 mg/ml. It is important that the final detergent concentration 20 mM if CHAPS is used or 30 mM (1.2%) if cholate is used.
- Then incubate 10 min on ice and centrifuge (4 °C, 20 min, Beckmann JA-20 (or Sorvall SS-34) fixed angle rotor, 18,000 rpm, 39,000×*g*). Reconstitute (see below) 20 µl from the supernatant (detergent-extract). Store the supernatant on ice. Half-life of glutamate uptake activity is variable: 30–120 min at 0 °C.

### Notes on Proteolysis of Glutamate Transporters

The both the N- and the C-terminus of EAAT2, but also the C-terminus of EAAT1, are fairly rapidly proteolysed after death (see: [42–44]. This means that the transporters become undetectable with most of the antibodies used to examine them in post mortal human tissue. In contrast, the C-terminus of EAAT3 degrades considerably slower. Further, the degradation rates differ between brain regions. At least some of the observations that there are differences in transporter distributions between human and rodents may be due to the fact that the various antibody epitopes degrade with different rates (for discussion and list of antibody epitopes, see: [44]).

Interestingly, the transport activity declines considerably slower after death than the immunoreactivity to the termini [43] in agreement with the observation that the central parts of the transporters degrade slowly [43, 44] and that transport activity does not depend on the termini (e.g. [45–47]). Thus, for isolation of EAAT2 from brain tissue, it is a good idea to “keep it cold and work fast”.

### Reconstitution

First, make the spin columns ready for sample application (see “Rapid gel filtration on spin columns” below). Then mix (up to 100 µl of) each fraction (to be tested) containing either 20 mM CHAPS or 1.2% (w/v) cholate with 150 µl standard reconstitution mixture (see above and Fig. 3). For reconstitution of GlyT2, 1.5% (w/v) cholate is preferred. Because of the high protein concentration in the detergent-extract (see above), only 20 µl is reconstituted. To compensate for volume, add 80 µl buffer with the above detergent concentrations to the 150 µl reconstitution mixture. Incubate at least 15 min on ice, but avoid excessive incubation times as the half-life of glutamate transport activity at this stage is about 2 h. The total volume of each sample is 0.25 ml. This is chosen to make it easy to load each spin columns with 0.2 ml.

#### Importance of Detergents

150 µl of the above reconstitution mixture is designed for reconstitution of glutamate and GABA transporters in a 100 µl sample containing either 1.2% (w/v) cholate alone or 0.1% (v/v) Triton X-100 with 0.9% (w/v) cholate or 20 mM CHAPS alone. As mentioned above, GlyT2 requires somewhat higher cholate concentrations. Both the volume of the sample and the amount of detergent are critical for formation of tight liposomes and thereby for transport activity. The highest transport activity (both GABA and glutamate) is observed using cholate as the only detergent. If the sample to be reconstituted has a different composition, dilute or add detergent to obtain the above concentrations.

During the centrifugation to remove the detergent, also the small molecular mass substances (i.e. salts and amino acids) present in the sample are lost (retained on the spin columns). Because detergent monomers are in equilibrium with larger detergent aggregates (micelles), they behave partly like small molecules and partly like high molecular mass substances during gel filtration. This means that they are incompletely removed. Cholate and CHAPS have relatively high CMCs implying that a relatively high percentage of the total is in monomer form and therefore can be removed by gel filtration. This is the reason why the amount of detergent present during reconstitution is critical. It must be enough detergent to solubilize and little enough for it to be removed.

We never tried dialysis. The reason was that our need was multiple small volume reconstitutions (to test many fractions). In contrast, Hannah Rahamimoff, who was working on the Na^+^–Ca^2+^-exchanger (she was one of Kanner’s neighbors), used dialysis in stead of gel filtration [48, 49]. Her need was reconstitution of large volumes as she was using transport specific fractionation of the liposomes [48].

It is interesting to note that none of the detergents tried in the 1990s could substitute for cholate in the reconstitution mixture. Some cholate must be present. However, as mentioned above, some of the newer detergents [41] have not been tried by us.

If the EAAT2 glutamate transporter protein has been exposed to Zwittergent 3–12, β-octylglycoside (CAS 29836-26-8) or sodium dodecyl sulfate (SDS; CAS 151-21-3) prior to reconstitution, there will be no glutamate transport activity. These detergents effectively and irreversibly inactivate these transporters [18, 50]. The glutamate uptake activity is also unstable in Triton X-100 (N.C. Danbolt, unpublished). [An additional challenge with Triton X-100 is the very low CMC.] In contrast, the GABA transport activity tolerates both Triton X-100 (CAS 9002-93-1) and β-octylglycoside. It is possible that the EAATs depend on trimeric structure to be active (for discussion see: [18]). Conversely, the GlyT2, which is also a dimeric protein [51] does also not tolerate β-octylglycoside for active transport and poorly Triton-X-100, suggesting there are specific requirements for every transporter.

#### Importance of Protein Concentrations

The maximum amount of protein to be reconstituted depends on the particular protein mixture and transporter to be assayed. It also depends on how important it is to obtain one transporter molecule (or oligomer) per liposome, and on how important it is to be sure that the uptake is linear with time. The liposomes have a variable diameter [23] and no system for renewal of the ion gradients driving the uptake process. Thus, the more transporter molecules there are in the same liposome, the quicker the dissipation of the ion gradients. For quantitative comparison of the uptake activity in various samples, it is therefore a good idea to keep both the transport activity and the incubation time relatively low. The routine has been to always keep the amount of protein per 150 µl reconstitution mix below 200 µg crude brain membrane proteins (about 4 mg intact wet tissue). This should be regarded as the upper limit for glutamate uptake and is usually too high (by a factor of 5 if the protein is very active).

### Some Special Reconstitution Cases

#### Reconstitution from Synaptosome Containing Sucrose Homogenate

Homogenize brain tissue in 10 volumes 0.32 M sucrose with 1 mM PMSF and 5 mM EDTA. Mix 83 µl of this homogenate (about 10 mg protein per ml) with 250 µl solubilization buffer (1.6 % (w/v) cholate, 900 mM NaCl, 50 mM NaPi pH 7.4). This gives a final cholate concentration of 1.2 % (w/v). Then incubate 5 min on ice and add 500 µl of the standard reconstitution mixture (see above). Then mix, incubate 15 min on ice and gel filter on spin columns to remove detergent and replace the reconstitution buffer with the desired internal medium.

#### Direct Reconstitution from Transfected Cells or Cell Membranes

The packed cells or cell membranes are solubilized in 4 volumes buffer containing 1.5 % (w/v) cholate, 500 mM NaCl and 100 mM sodium phosphate buffer pH 7.4 (to give 1.2 % (w/v) cholate in the final mixture). After incubation (10 min, 0 °C), the mixture is centrifuged (18.000 rpm, 39.000 x g, 20 min, 4 °C). The supernatant is mixed with 1.5 volumes reconstitution mixture, incubated (15 min, 0 °C) and gel filtrated on spin columns (see below) to remove detergent and replace the reconstitution buffer with the desired internal medium.

#### Direct Reconstitution from Intact Brain Tissue

The tissue or packed cell membranes are solubilized in 25 volumes buffer containing 1.25% (w/v) cholate, 500 mM NaCl and 100 mM sodium phosphate buffer pH 7.4 (to give 1.2% (w/v) cholate in the final mixture) and 1 mM phenylmethanesulfonyl fluoride (PMSF; CAS 329-98-6) as protease inhibitor (optional). After incubation (10 min, 0 °C), the mixture is centrifuged (Beckmann JA-20 fixed angle rotor, 39,000×*g*, 18,000 rpm, 20 min, 4 °C). The supernatant is mixed with 1.5 volumes of the above reconstitution mixture, incubated (15 min, 0 °C) and gel filtrated on spin columns (see below) to remove detergent and replace the reconstitution buffer with the desired internal medium. This means that the reconstitution mixture contained about 15 times more lipids than the endogenous lipids present in the rat of mouse brain extracts.

### Notes on Solubilization of EAATs for Western Blotting

There has been some confusion in the literature with respect to how to best to detect the EAAT-type of proteins by Western blotting (see also: [52]).

When quantifying EAATs by Western blotting, it is desirable to have all of the EAAT-protein in monomer form, and not as a mixture of monomers and oligomers. Both rat [18] and bacterial [53, 54] EAATs exist in the membranes as trimers. The subunits are non-covalently connected. The non-covalent association is apparent when fresh whole tissue is solubilized by direct homogenization in (10 mg/ml) SDS. In that case, only monomers are seen on Western blots. If the membranes or tissue homogenates have been allowed to oxidize, then cross-links are formed, but these are reversible by inclusion of 30 mM 1,4-dithiothreitol (DTT; CAS 3483-12-3) to the sample before loading on the electrophoresis gels. The confusing phenomenon is that SDS-resistant complexes are formed if the lipids are extracted with a mild detergent (e.g. Triton X-100, cholate, etc.) before SDS is added. In that case, the glutamate transporter subunits gradually (temperature dependent) get entangled in each other in such away that they cannot be separated any more [52, 55]. Thus, avoid mild detergents and buffers that contain them. The commonly used “RIPA Lysis and Extraction Buffer” (Cold Spring Harbor Protocols) is one example of buffer to avoid. Also note that the antigen preadsorption test can lead to inaccurate assessment of antibody specificity [56].

Another point, water soluble proteins present in the tissue or in cultured cells, can interfere with the binding of the transporter proteins to the nitrocellulose membranes [52, 57, 58]. In fact, addition of water soluble proteins from non-transfected HEK293T cells to the sample can result in complete failure to detect the BGT1 (Slc6a12), EAAT2 (Slc1a2) and EAAT5 (Slc1a7) transporters. Further, it is common to find a weak cross-reactivity with proteins of the same molecular mas as EAAT2 in the water soluble fraction (see: [52, 59]). The strongest (specific) signal on Western blots is thereby obtained when the tissue or cultured cells are first homogenized in water (with 5 mM EDTA and 1 mM PMSF), centrifuged to collect the membranes (20,000×*g*, 15 min, 4 °C) and then solubilized in SDS (5–10 mg/ml) as this procedure will remove the water soluble proteins that interfere with the binding of the transporters. An additional advantage is that this will also remove potassium ions which precipitates SDS (as potassium dodecyl sulfate is insoluble) and thereby make the mixture viscous. DNA will also give a high viscosity, but a quick sonication breaks down the DNA sufficiently to eliminate that problem. It should be noted that SDS will inactivate the EAATs (as well as the GlyTs and GATs), so if the tissue is also to be assayed for transport activity, then the homogenate should be aliquoted before addition of SDS. The transporter proteins are stable as long as they are sitting in the native membranes and proteolysis is avoided. Thus, the membranes containing the membrane proteins can be frozen and stored at − 80 °C without loss of transport activity.

### Internal Medium

Because of the efficiency of the rapid gel filtration described below, the salts present in the reconstitution mixture and in the protein extract will be removed. It is therefore the medium that the gel filtration columns are equilibrated with that becomes trapped inside the liposomes when they form and thereby becomes the internal medium. The medium used as the standard internal medium in the Danbolt lab for measurement of net uptake of glutamate and GABA consists of 0.12 M KPi buffer pH 7.4 (0.12 M with respect to phosphate) and 1% (v/v) glycerol. However, this medium can be modified as desired (e.g. [16, 19, 25]). For instance, rubidium or cesium can be used in-stead of potassium and the concentrations can be higher. The peak of glutamate transport activity in this assay is seen with about 600 mM potassium (N.C. Danbolt, unpublished data). Alternatively, if heteroexchange is to be studied, then sodium can be used in-stead of potassium. If so, a transporter substrate, e.g. l-glutamate (l-Glu) or d-aspartate (d-Asp), must be added to the internal medium (see e.g. [19]; also see Fig. 5 in [55]).

### Rapid Gel Filtration on Spin Columns

As already explained, the reconstitution depends on removal of the detergent (cholate) as the liposomes form spontaneously when this happens. This can be done in several ways. Kanner introduced rapid gel filtration on spin columns (Figs. 4 and 6f). The rapid removal of the detergent allows quick inclusion of the transporter protein in a lipid environment. This is protective for the transport activity of the protein under purification. Besides, if detergent is removed rapidly, it results in a more homogeneous population of small vesicles [60].

The columns described here are useful for rapid gel filtering of samples in general. For instance, substances that interfere with protein measurement or electrophoresis can be quickly removed. (If the samples are intended for protein measurement with the method of Lowry [61], then the Sephadex must be carefully washed to remove dextran fragments as they interfere.) Our main use of them, however, has been to remove detergents during reconstitution of glutamate and GABA transporters, and for washing of liposomes (replacing the external medium). The efficiency of the spin columns can be tested by adding radiolabeled small molecules, i.e. amino acids, to the sample. The 1 ml columns reduce added l-[^3^ H]glutamate about 5000 fold (N.C. Danbolt, unpublished data [17]).

Procedure: Swell Sephadex G-50 fine (for proteins larger than 40 kDa) in the desired internal or external medium (1 g in no less than 15 ml) overnight in a refrigerator. Pack the swelled gel in 1 ml plastic syringes (Fig. 4) from which the pistons have been removed and the outlets closed by ordinary cotton fiber. Centrifuge the columns for 2 min to remove the void volume. This can be done several hours prior to use (N.C. Danbolt, unpublished data) provided they are kept at 4 °C. Load 0.2 ml sample onto each column. It is important that the sample is added quickly so that the gel swell and thereby prevent the sample from flowing between the compacted gel and the cylinder wall. After allowing the fluid to sink into the gel, the columns are centrifuged again.

Obviously, the centrifugation speed is important. The centrifuge used in Danbolt’s laboratory is a Heraeus-Christ Minifuge-T. With this centrifuge it works well to remove the void volume by centrifugation 1200 rpm (240×*g*) for 2 min at 4 °C. After applying the sample (0.2 ml) and allowing it to sink into the gel, the columns are centrifuged again at 1740 rpm (500×*g*) for 2 min at 4 °C. The conditions are adjusted using phenol red and Blue-Dextran 2000 (CAS 87915-38-6) as low and high molecular mass marker, respectively. When the conditions are properly adjusted, then the low molecular mass compound should be retained, while the other should pass through (Fig. 4).

After reconstitution (detergent removal) the liposomes are suspended in the internal medium: the internal medium is also on the outside. In fact, in 20 µl liposome suspension, only about 0.5 µl is actually inside the liposomes (N.C. Danbolt, unpublished). Therefore, if it is important not to contaminate the external medium with the internal medium, then the liposomes can be washed by another gel filtration as above, but with columns equilibrated in external medium rather than internal medium. One example of a situation when this is important, is when studying heteroexchange with liposomes loaded with 20 mM glutamate. In that case the liposomes had to be washed twice by centrifugation to remove all of the l-Glu present on the outside (see: [19]). If the liposomes are loaded with a cesium containing internal medium and suspended in a sodium containing external medium for some time before the substrate is added, then there will be a gradual loss of transport activity due to dissipation of the ion gradients, but some uptake activity may still be present after several hours.

### Amino Acid Transport—Uptake in Liposomes

**Fig. 5:**
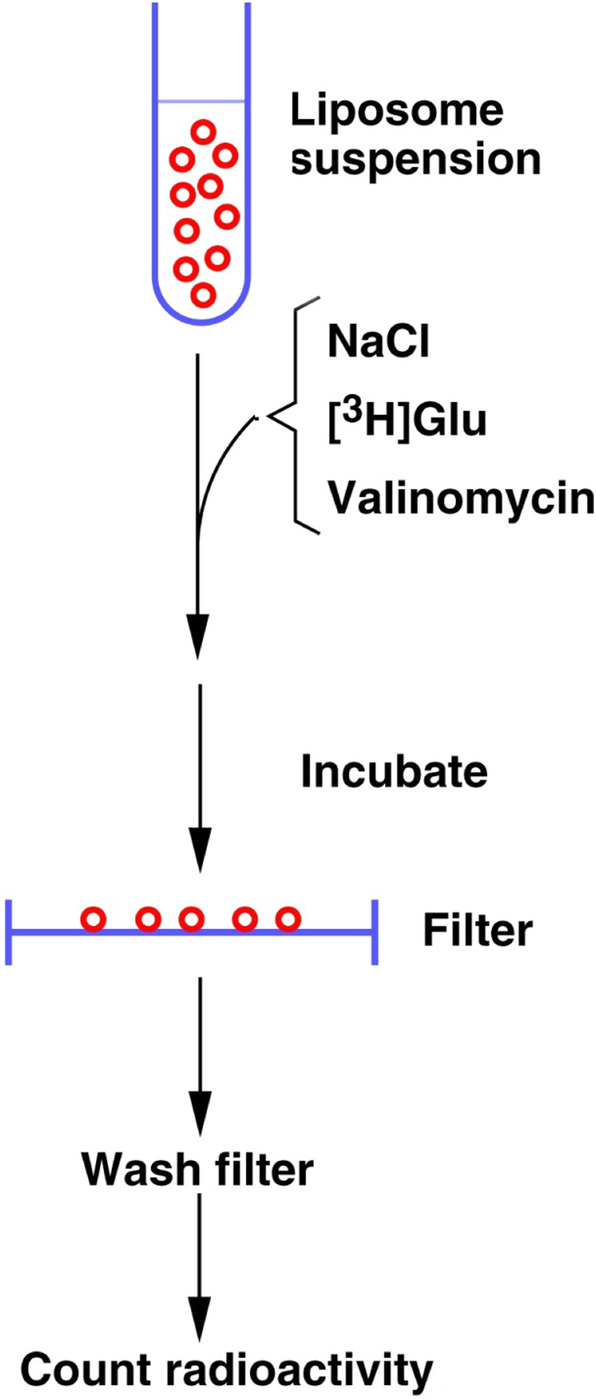
Schematic illustration of the uptake assay. The liposome suspension (e.g. 20 µl) is diluted into an external medium (e.g. 500 µl) containing radioactively labeled substrate and incubated. The reaction is stopped by dilution in ice-cold medium without substrate and quickly filtered to isolate the liposomes. After quick rinsing of the filters with more of the ice-cold medium, the radioactivity retained in the trapped liposomes is measured. Copyright: Neurotransporter.org; reproduced with permission

The uptake reaction is started by mixing the liposome suspension (20 µl) with the desired external medium (500 µl) containing a radioactive substrate (Fig. 5). Like the internal medium, the external medium can be altered as desired provided that the internal and the external media have the same osmotic strength. Obviously, the media must not contain detergents or other substances that compromise the liposome membranes. The standard external medium used in Danbolt’s laboratory (www.neurotransporter.org) in combination with the standard internal medium (0.12 M KPi with 1% (v/v) glycerol) is simply 0.15 M NaCl with 1% (v/v) glycerol plus 1.4 µCi (about 50 nM) radioactive substrate (e.g. d-[^3^ H]aspartate, or l-[^3^ H]glutamate or [^3^ H]GABA) and 3 µM valinomycin. Valinomycin (CAS 2001-95-8) from *Streptomyces fulvissimus* allows K^+^ to pass through the membrane down its concentration gradient creating an internal negative membrane potential relative to the outside [62, 63]. Thus, although valinomycin is not a channel, the functional consequences of its action are the same as the operation of an ion channel much like the potassium channels causing the negative membrane potential in mammalian cells. The negative membrane potential stimulates transport of glutamate, GABA and glycine, which are electrogenic. In fact, the stimulation of transport by valinomycin is higher in proteoliposomes than in membranes. This is apparently due to a lowered potassium permeability caused by the greater phospholipid/protein ratio present in reconstituted systems [14]. We prepare stock solutions of valinomycin (10 mM) in dimethyl sulfoxide (CAS 67-68-5). This solution is stable for a couple of years if stored at – 20 °C.

The external medium should be sterilized by filtration (e.g. through Millex-HA, 0.45 μm pore size) before addition of valinomycin and labeled substrate to eliminate background caused by bacteria accumulating radioactive glutamate.

Addition of 2.8 µM of nigericin (CAS 28643-80-3) is a good negative control. Nigericin is produced by *Streptomyces hygroscopicus* and is toxic. It is a carboxylic ionophore and catalyzes an electroneutral exchange H^+^ with an alkali metal cation. When nigericin is added to a cell, a transient internal acidification will occur because K^+^/H^+^ exchange is faster than the Na^+^/H^+^ exchange. However, the latter process will also take place leading to a dissipation of the gradients [62]. We prepare stock solutions (5 mM) in dimethyl sulfoxide (CAS 67-68-5). These solutions are also stable for a couple of years if stored at − 20 °C.

Other suitable negative controls that can be added to the external medium comprise dihydrokainic acid (CAS 52497-36-6; a selective EAAT2 competitive blocker) [64, 65], or derivatives of the nonselective EAAT blocker DLthreo-beta-benzyloxyaspartate (DL-TBOA; CAS 205309-81-5 [66]) such as (3S)-3-[[3-[[4-(trifluoromethyl)benzoyl]amino]phenyl]methoxy]-l-aspartic acid (TFB-TBOA; CAS 480439-73-4 [67]). Presently, for the GAT1 transporter, the specific inhibitors 1-(4,4-diphenyl-3-butenyl)-3-piperidinecarboxylic acid hydrochloride (SKF-89,976 A; CAS 85375-15-1) or 1-(4,4-diphenyl-3-butenyl)-1,2,5,6-tetrahydro-3-pyridinecarboxylic acid (SKF 100,330 A (CAS 85375-88-8) at 30 µM [68] could be used as a negative control. In addition, the GlyT2 transporter can now be blocked by 1 µM O-[(2-benzyloxyphenyl-3-flurophenyl)methyl]-l-serine (ALX-1393) or N-[[1-(Dimethylamino)cyclopentyl]methyl]-3,5-dimethoxy-4-(phenylmethoxy)benzamide hydrochloride (ORG25543; CAS 495076-64-7) [69].

The uptake reaction is usually started by adding 20 µl of the liposome suspension to 500 µl of the external medium containing sodium, radioactively labeled substrate and ionophores as desired. Accurate pipetting of the liposomes is essential. This is difficult because the liposome suspension sticks to the pipette tip both on the outside and inside. Thus, the tip should not be put deep into the liposome suspension to avoid contamination on the outside and care must be taken to empty it properly by pipetting in and out a few times.

The transport reactions are terminated by the dilution of 2 ml ice-cold external medium (without substrates) and rapid filtration through Millipore HAWP filters (0.45 μm pores; nitrocellulose-cellulose acetate). The incubation time for routine determination of glutamate and GABA uptake activities have been, respectively, 70 s and 5–7 min. But for studies of kinetics, the incubation time has been 2 s (e.g. [19]). To start and stop reactions within 2 s, a metronome was used: start the uptake reaction on a click, wait one click and stop on the next click. However, for the glycine transporter whose transport activity in nervous tissue is about 7 to 10 fold lower than that of the glutamate transporter, the transport time had to be increased during purification in order to detect the activity despite losing some quantification accuracy [21].

The filters are rinsed with ice-cold washing solution three times and then dried (room temperature). Finally, they are put into counting vials together with 0.1 ml water and 3.5 ml Filter-Count (Packard) for liquid scintillation counting. It is important to give the radioactivity sufficient time to come into solution before counting.

Note that the pores of the filters are larger than the liposomes. Retention of the liposomes by the filters is therefore dependent on binding and not, or to a lesser degree, on mechanical retention. This implies that the binding sites on the filters may be saturated. Thus, when pouring the diluted liposomes onto the filters, make sure to do this quickly so that the whole surface of the filter is utilized. If the fluid is only passed through a part of the filter, fewer liposomes will be retained because the binding sites will be saturated on this part of the filter. This also may explain why there is no point in reducing the pore size of the filters from 0.45 to 0.22 μm.

## Scientific Impact of This Reconstitution Method

### Molecular Identification and Cloning of GABA, Glutamate and Glycine Transporters

In the 1970s, at the time when Kanner started to study GABA and glutamate uptake, it was clear that the GABA uptake was independent from that of glutamate uptake, and that the two uptake processes required energy. But beyond that, little was known. The processes were assumed to be due to proteins, but even that was an assumption! Thus, there were no antibodies, no PCR-probes and no high-affinity ligands that could be used to detect these transporters. The only way to identify them was by their transport functions. To complicate it further, membrane proteins can only be purified if they can be taken out of the membrane they are sitting in as those membranes also harbor other membrane proteins. But when the lipid membranes are dissolved and the proteins are free in solution surrounded by detergent molecules, there are no compartments and transport has no meaning. Thus, to monitor a purification process (as illustrated in Fig. 2), it was necessary to have a method whereby the membrane proteins could be reconstituted into artificial cell membranes (Fig. 3) so that each purified fraction could be tested for content of molecules with ability to transport (Fig. 5). However, this would only work as long as the solubilization and chromatographic procedures were so gentle that transporters were not irreversibly denatured. Further, the literature on protein purification concerned water soluble proteins, implying that the purification of transporter proteins was uncharted territory.

**Fig. 6:**
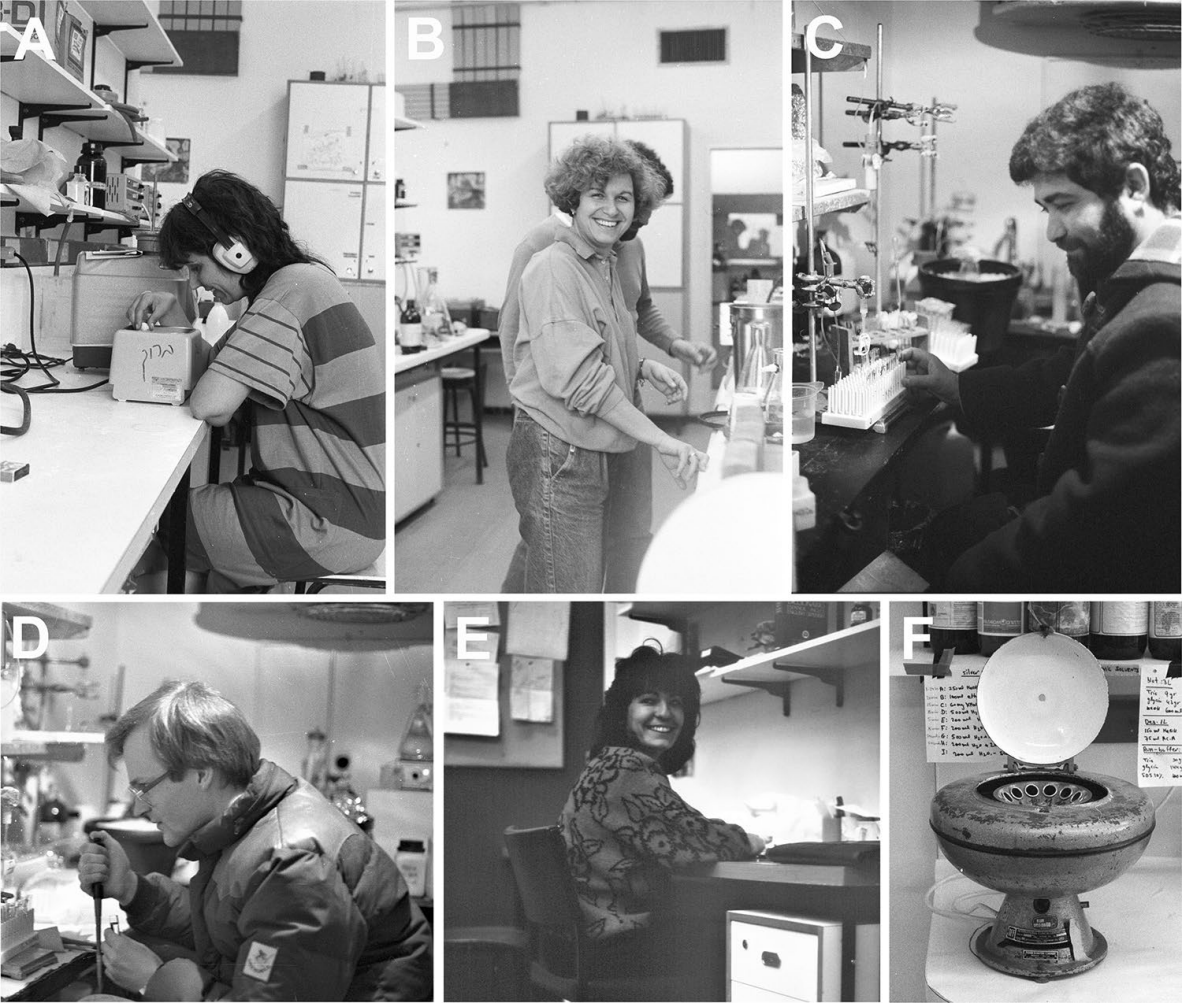
The environment in Kanner’s lab and in the department as a whole was very friendly and very creative. Further, “out of the box” thinking was stimulated by a multicultural encounter between people from different backgrounds and countries. **a** Annie Bendahan is here photographed while sonicating lipids for reconstitution of GABA transporters. She worked with Kanner for 30 years from 1981 and generated or helped generating many of the most important data from the laboratory. **b** Rodica Radian-Gordon who isolated GABA transporter 1 [14, 20] is here depicted in the laboratory assisted by her husband, Alexander Gordon (Alex). Her career path has been extraordinary. She is now (since 2019) the Israeli Ambassador to Spain after having been Israeli Ambassador to several other countries! **c** Alex obtained a partial purification of glutamate transporters using lectin (WGA; Wheat germ agglutinin) affinity chromatography [15]. Here he is photographed when running a WGA-column in the cold room. **d** Niels Christian Danbolt learned from Alex, modified both the WGA-chromatography and the reconstitution procedure and eventually managed to isolate active glutamate transporters using three chromatographic steps [13]. Here he is photographed in the cold room (by Michela Tessari [49]). **e** Beatriz López-Corcuera visited Kanner’s lab to get help with the isolation of glycine transporters. She also succeeded [21, 35]. **f** This is the old tabletop centrifuge used for all of the reconstitutions in Kanner’s laboratory. *This clearly illustrates that intelligence is more important than fancy equipment!* Copyright: Neurotransporter.org; reproduced with permission

Despite the challenges, the use of reconstitution of transport activity to monitor the purification led to the successful isolation of a GABA transporter [14, 20] by Rodica Radian-Gordon and Annie Bendahan in Kanner’s laboratory (Fig. 6a and b). Based on sequences from the purified protein, Kanner, with the help of Henry A. Lester and Nathan Nelson, managed to clone the first neurotransmitter transporter [70] now known as GAT1 (Slc6a1). The sequence was novel and not related to any proteins known at the time. Using antibodies to the purified protein, GAT1 was found to be primarily neuronal although astroglial expression was also found in some regions, particularly in the thalamus [71] (for review see: [72]).

Shortly after the cloning of GAT1 [70] Susan G. Amara used expression cloning and identified the noradrenaline transporter (NET; Slc6a2 [73], and then found that it had substantial similarities with GAT1. Thus, it was clear that a new gene family had been discovered. Based on sequence homology, several other transporters were identified [74]. Surprisingly, no glutamate transporter was identified this way.

Although GAT1 is an abundant isoform [14], early biochemical investigations showed that there had to be more than one GABA transporter because GABA uptake by astrocytes and neurons could be distinguished pharmacologically [75, 76]. Indeed, Kanner and co-workers were able to separate GAT1 from GABA transporters with a different substrate selectivity by means of ammonium sulfate fractionation of solubilized brain proteins [77, 78]. In fact, altogether four GABA transporters have now been identified: GAT1 (Slc6a1) GAT2 (Slc6a13; mGAT3), GAT3 (Slc6a11; mGAT4) and BGT1 (Slc6a12; mGAT2). All the four subtypes are present intracranially [70, 79–84]. Using knockout mice we have shown [58, 85, 86] that the major isoforms in the brain are GAT1 and GAT3 in agreement with Kanner’s biochemical data [77, 78] as we found that GAT2 and BGT1 are primarily expressed in the liver (in the hepatocytes), in the kidney and in the leptomeninges [58, 85–88].

Kanner’s reconstitution procedure was adapted to work for glutamate [15] and glycine transporters [35]. This led (Fig. 6c and d) to the successful isolation [13, 89] and cloning [50] of the major glutamate transporter now known as EAAT2 (GLT-1; Slc1a2). It also (Fig. 6e) lead to the isolation of the GlyT2 (Slc6a5) glycine transporter [21, 35] as well as to the development of the first antibodies to the mentioned transporters and thereby to the localization of the proteins (e.g. [71, 89–92]).

The purification of GlyT2 revealed for the first time the existence of two glycine transporters. The purified transporter activity was pharmacologically different from that measured in plasma membranes, which was mainly sensitive to sarcosine (N-methyl-glycine) whereas GlyT2 was resistant [21]. Afterwards, the sarcosine-sensitive GlyT1 transporter was cloned [93].

While GlyT2 [94] turned out to be a new member of the same family as GAT1 and NET, the sequence of EAAT2 was novel and not related to any proteins known at the time with the exception of some similarities with a bacterial dicarboxylate transporter [50]. It was clear that this was another novel gene family [95] when two other glutamate transporters were identified. These two were identified at the same time by three other research teams using very different approaches: rat EAAT1 (GLAST; Slc1a3 [96, 97]) and rabbit EAAT3 (EAAC1; Slc1a1 [98]). The human and rat counterparts were quickly cloned [64, 99]. Two additional glutamate transporters, EAAT4 (Slc1a6) and EAAT5 (Slc1a7), were found later [100, 101].

The two transporter families, subsequently named SLC6 and SLC1, include non-homologous transporters [1]. The SLC6 family comprise 20 different Na^+^-and Cl^−^-dependent transporters including the neurotransmitter transporters for GABA, glycine, and monoamines, which are 12 transmembrane domain proteins with intracellular N- and C-termini [102–106], whose 3D structure have been modeled based on prokaryotic and eukaryotic crystals[107–110]. On the other hand, the SLC1 family includes as already mentioned above, the five Na^+^–K^+^ and H^+^-dependent glutamate (excitatory amino acid) transporters (EAATs) and two neutral amino acid transporters [55, 111–113]. These transporters are functionally and structurally unrelated to the SLC6 family and have 8 transmembrane domains with two membrane reentrant loops and N- and C-termini in the cytoplasm, whose 3D structure has been also modeled based in crystallized structures [41, 53]. In the two families, however, the transmembrane domains organize in a different although equivalent functional way, to orient the central substrates-binding pocket to gain alternatively access to either side of the membrane via conformational changes [114].

### Uncovering of the Driving Forces of Neurotransmitter Uptake

Early studies had shown that neurotransmitter transport was sensitive to ouabain (an inhibitor of the Na^+^K^+^-ATPase), and that the transport was impaired when the energy metabolism was compromised [115–119]. This led to the hypothesis that the electrochemical gradients, generated primarily by Na^+^K^+^-ATPase, also drive neurotransmitter uptake [115, 116, 120, 121].

In order to advance, it was necessary to be able to study the properties of the proteins contained in plasma membranes without interference from intracellular metabolism and subcellular organelles. Kanner therefore introduced the use of isolated plasma membrane vesicles, in which he could artificially create ion gradients [7]. These studies revealed that, in addition to Na^+^, the membrane potential itself mattered and that other ions were also required. For instance, translocation of inhibitory transmitters GABA and glycine depends on the presence of external Cl^−^ [35, 122–124], while glutamate uptake requires the presence of internal K^+^ [4, 5, 125]. In parallel, other investigators found that the transport generates pH changes [126, 127]. By using whole cell clamping on either a CHO cell line selected for low endogenous glutamate uptake or *Xenopus laevis* oocytes expressing transporter proteins, it was concluded that glutamate transport catalyzed by EAAT1-3 is accompanied by cotransport of three Na^+^ and one H^+^ and counter-transport of one K^+^ [128–131] as well as water [132].

These studies predicted that extracellular glutamate and GABA can be maintained in the nanomolar range [128, 129], provided that there is a sufficient number of transporter molecules. This was shown to be the case [133, 134] as quantitative immunoblotting of brain tissue extracts compared with known amounts of purified glutamate transporters revealed that the EAAT2 protein represents about 1 % of the total forebrain protein [57, 135–137]. Ambient glutamate both in- and outside the synaptic cleft was further experimentally examined by utilizing NMDA receptors as glutamate sensors in acute brain slices, and is found to be near 25 nM [138–140].

The stoichiometry for the translocation cycle catalyzed by the GABA transporter has been debated. Keynan and Kanner found it to be between 2 and 3 Na^+^ ions as well as a Cl^−^ ion [141]. Three other research teams concluded that it was 2 Na^+^ ions [142–144], while the most recent paper on the topic concludes that it is 3 Na^+^ ions and 1 Cl^−^ ion per GABA molecule [145, 146]. Quantification of GABA transporters similarly revealed very high levels of GAT1 [147] in agreement with the observations that mice lacking GAT1 display behavioral abnormalities [148–155].

### Chloride Channels Inside Glutamate Transporters

The first indication of uncoupled anion fluxes in connection with glutamate transport was the observations, by Kanner, of the effect of external chloride ions on glutamate transport into brain membrane vesicles (see Fig. 2 in [4]). About ten years later, it was noted that vertebrate photoreceptor cells have a dual character being partly transporters and partly ion channels [156–158]. The detailed characterization of EAATs by heterologous expression in *Xenopus oocytes* and various mammalian cells revealed a reversible and thermodynamically uncoupled chloride flux which is activated by glutamate transporter substrates [100, 128, 159–162].

The different transporter subtypes display varying degrees of this thermodynamically uncoupled glutamate-activated chloride flux (EAAT4, EAAT5 > EAAT1 > EAAT3 > > EAAT2: [163, 164]. EAAT4 and EAAT5 may actually function more as inhibitory glutamate receptors than as transporters [136, 165, 166].

Site-directed mutation analysis strongly suggest that the chloride permeability is within glutamate transporter proteins themselves and further revealed separate molecular determinants associated to the two functions: The second, fifth and seventh transmembrane domain are involved in this chloride permeability [167–169] while the glutamate translocation domain is composed of transmembrane domains 3, 6–8 and reentrant loops HP1 and HP2 [170].

However, uncertainties have remained due to the presence of other transporters and ion channels in the plasma membranes. A “clean” approach to this problem is to use artificial cell membranes (liposomes). When reconstituting the purified recombinant bacterial glutamate transporter protein, Glt(Ph), an uncoupled Cl^−^ conductance was detected [161] because Cl^−^ affected the rate of substrate uptake by changing the membrane potential. In parallel, when resolving the question of the paradoxically high glutamate uptake in axonal terminals (see below), we also noted [19] anion channel activity in the mammalian EAAT2 (Fig. 11), which is the least Cl^−^ permeable subtype. Little is yet known about the functional importance of the uncoupled anion flux, but it may play a role during astrocyte maturation and in response to cerebellar activity [171] and abnormalities in EAAT-associated anion currents may cause glial apoptosis [172].

### Interaction of Lipids with Neurotransmitter Transporters

Reconstitution of purified GABA transporter preparations directly into asolectin liposomes resulted in low transport activity. A dramatic increase of GABA and glutamate transport activities was seen when the liposomes were supplemented with brain lipids. The effect of brain lipids appeared, at least in part, to be due to cholesterol [31]. Recent studies of bacterial secondary transporters provide a structural evidence for functional interactions of lipids with the catalytic core in controlling the conformational equilibrium [173–175].

Glutamate uptake appeared less dependent on brain lipids, but addition of brain lipids or only cholesterol also increased reconstituted glutamate uptake. In support of the notion, depletion of membrane cholesterol by pharmacological and genetic means resulted in reduced glutamate uptake [32, 176, 177].

### The Present Reconstitution Assay has been Used to Distinguish Between Indirect and Direct Effects on the Transporter Proteins

Arachidonic acid (as well as other cis-polyunsaturated fatty acids) was reported to inhibit several sodium coupled amino acid transporters including the uptake systems for glutamate, glycine and GABA [178–188]. As all these studies were performed on complex preparations such as synaptosomes or intact cultured cells (neurons and astrocytes), we tested the effects of arachidonic acid and several other fatty acids and methyl esterster, on glutamate transporters after reconstitution in artificial cell membranes (liposomes) devoid of all other enzymes and signaling molecules [17]. This confirmed that arachidonic acid inhibits EAAT2, and showed that the effect was due to direct action on the transporter itself rather than an effect through other mechanisms or via the phospholipid membrane.

It was noted in the mid 80’s that glutamate uptake is sensitive SH-group oxidants (for references, see: [189]). The oxidizing and reducing agents also had effect on reconstituted glutamate transporters and this may suggest that the glutamate transporters possess an SH-based redox regulatory mechanism [190, 191].

It was noted that a compound from *Parawixia bistriata* spider venom enhanced glutamate uptake by synaptosomes [192]. To determine if the novel compound acted directly on the glutamate transporters rather than acted indirectly via other mechanisms, it was tested on liposomes containing EAAT2 and found to enhance glutamate uptake there too [193].

### Neuronal Glutamate Uptake in Axon Terminals

The question of whether nerve terminals are able to take up glutamate to a physiologically significant degree has been controversial since the end of the 1970s (for review see: [55, 194]).

Using d-aspartate (d-Asp) and anti-d-Asp antibodies in combination with electron microscopy we found that there are glutamate transporters, not only in astrocytes, but also in axon-terminals because they concentrate d-Asp sodium dependently and can be inhibited by excess l-Glu [195]. This confirmed previous studies based on autoradiography of tritium labeled substrates [196, 197]. We followed up on this and found [198] that the uptake into axon-terminals was sensitive to inhibition by dihydrokainate (an EAAT2 selective blocker; CAS 52497-36-6: [64, 65]) and was absent [198] in the EAAT2-deficient mice [199]. Indeed, in agreement with earlier detection of EAAT2 mRNA in hippocampal CA3 pyramidal cells [200–202], EAAT2 protein was detected in putative glutamatergic axon-terminals in hippocampus CA1 albeit at a level about 10 times lower than in astrocytes [198]. The EAAT2 protein was neither detected in cell bodies nor in dendrites [198] in contrast to EAAT3 [137] which is selectively expressed in cell bodies and dendrites (not in glia and not in axon-terminals). The lack of detectable d-Asp uptake in dendritic spines [198] is in agreement with the observation that the tissue content of EAAT3 is 100 times lower than that of EAAT2 [137].

The most surprising finding was not that axon-terminals express EAAT2 as that had been expected (for discussion and references see e.g.: [87, 194, 203]). The biggest surprise was that the d-Asp uptake into axon-terminals in hippocampal slices was as fast as that into astroglia despite being fully dependent on EAAT2 which was present in astroglia at about ten times higher levels than in axon-terminals [198]. In crude synaptosome containing homogenates (fresh brain tissue homogenized in 0.32 M sucrose without centrifugation) an even higher proportion of the accumulated substrate was found in terminals [198]. Together, these data showed that the rate of uptake into terminals did not fit with the distribution of EAAT2 (more uptake into terminals despite lower EAAT2 densities).

The reconstitution method described here was important in resolving this conundrum [194] in two ways: Firstly, it was instrumental in determining the relative rates of net uptake versus heteroexchange as explained below. Secondly, it was necessary in order to understand the data obtained after conditional deletion of EAAT2.

By floxing the EAAT2 (Slc1a2) gene (i.e. B6.Cg-Slc1a2tm1.1Ncd/J; RRID:IMSR_JAX:026619 [59, 204] it became possible to selectively eliminate EAAT2 from neurons or from astroglia. Deletion of EAAT2 selectively in astroglia in mice [204, 205] led to increased synaptic excitability, spontaneous seizures and a high mortality similar to that of the conventional (global) EAAT2 knockout mice [199]. This suggested that astroglial EAAT2 is the main protector against excitotoxicity in agreement with the facts that EAAT2 is the most abundant glutamate transporter [18, 135] and that most of the EAAT2 protein is in astroglia [89, 90, 206].

**Fig. 7:**
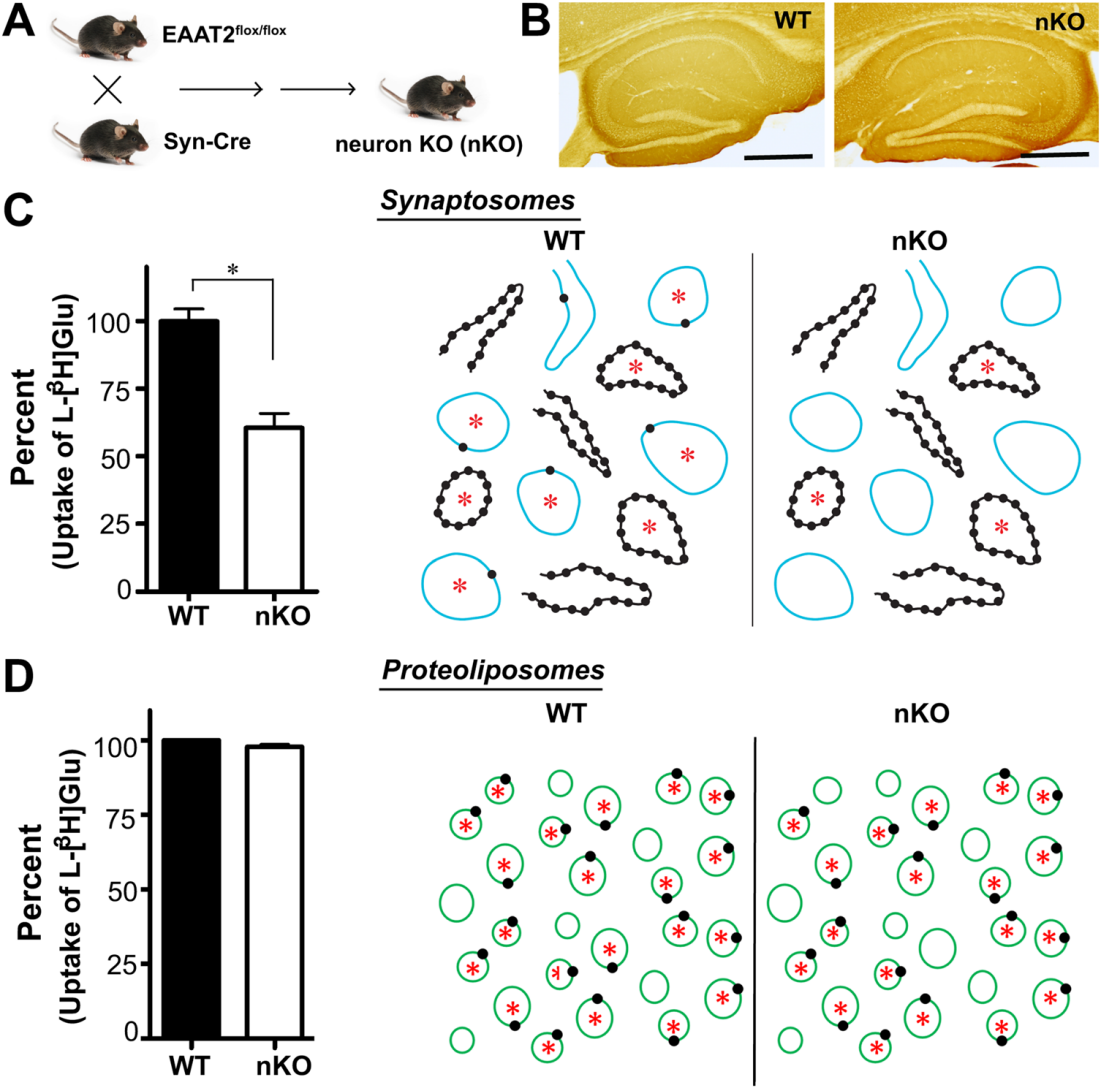
Astroglial EAAT2 is silent in a synaptosome containing tissue homogenate, but not if reconstituted into artificial cell membranes (proteoliposomes). **a** Mice homozygote for the floxed EAAT2 (Slc1a2) gene (i.e. B6.Cg-Slc1a2tm1.1Ncd/J; RRID:IMSR_JAX:026619 [59]) were bred with mice harboring one flox-EAAT2 allele and one Cre allele (B6.Cg-Tg(Syn1-cre)671Jxm/J}; RRID:IMSR_JAX:003966 [207]). This breeding yielded both mice with normal EAAT2 expression (wild-type; WT) and mice lacking EAAT2 (nKO) in neurons (for details see [9]). **b** Western blots (not shown) and immunocytochemistry showed that deletion of the EAAT2 gene in neurons had only minor impact on the total tissue content of EAAT2 protein (tntibody to EAAT2: RRID:AB_2714090; 44). **c** Forebrains from the nKO mice were homogenized in 0.32 M sucrose and assayed for glutamate uptake. The homogenates from the nKO mice had considerably lower glutamate uptake activity (left). The reason may be that (right) many of the astroglial cell fragments do not properly reseal so that only those indicated (red asterisk) accumulate glutamate. Consequently, all the EAAT2 transporter molecules (black dots) in the other fragments are unable to contribute to the uptake, and loss of the low number of transporters on neuronal membranes (blue lines) results in a major reduction in uptake activity. **d** When all the transporters are taken out of their original membranes and reconstituted into artificial ones (liposomes, green lines), then all transporters, regardless of whether they originate from astrocytes or neurons, have the same possibility to contribute to the uptake. In that case, loss of the few neuronal EAAT2 transporter molecules do not matter much. (Copyright: Panels B, C and left part of D are reproduced from Figs. 1 and 2a and d in Zhou et al. [9])

As expected from the above [198], deletion of the EAAT2 gene in astrocytes dramatically reduced the total amount of EAAT2 protein in the brain tissue as determined by Western blotting and by immunocytochemistry [204]. However, in agreement with the electron microscopy studies above [198], most of the uptake activity in crude synaptosome-containing tissue homogenates was still present. On the other hand [204], deletion of the EAAT2 gene in neurons (Fig. 7a) had only marginal effects on the total EAAT2 levels as determined by Western blotting and immunocytochemistry (Fig. 7b) in agreement with the notion that only a few percent of the EAAT2 protein is in neurons. Nevertheless, deletion in neurons had a major impact on the uptake activity observed in the crude synaptosome preparations (Fig. 7c) in agreement with the electron microscopy studies above [198].

The physiological roles of the neuronal EAAT2 need further studies, but it is already clear that it does have physiological roles [9]. The phenotypes so far identified from the neuronal EAAT2 knockouts suggested involvement of glutamate homeostasis and mitochondrial function [9, 208, 209], blunted locomotor response to acute AMP administration [210]; alteration in expression of cannabinoid receptors, preproenkephalin and PDE10A [211].

### Why is EAAT2 in Terminals so Easily Detected?

As explained above, when adding a labeled or artificial EAAT2 substrate to a synaptosome containing brain homogenate or to a fresh brain slice, about half of the added substrate ends up in terminals even though they only express a few percent of total EAAT2.

One factor of particular importance for tissue homogenates is the ability of the cellular fragments to for tight compartments. When the connection between an axon and a terminal is broken, then the diameter of the rupture is likely to be far smaller than the diameter of the terminal. In contrast, when a small branch of an astrocyte is torn off, then the diameter of the rupture may be as large as the diameter of the fragment. Thus, terminals are expected to have a higher probability of resealing, and thereby form structures with ability to accumulate substrate [9]. If so, then the astrocytic EAAT2 is mostly silent in the homogenate simply because they are sitting in the membranes of leaky compartments as illustrated in Fig. 7c. On the other hand, if all of the EAAT2 protein is solubilized and reconstituted so that all the transporter molecules, regardless of origin, have the same probability of being in a membrane of a non-leaky compartment, then astroglial EAAT2 should contribute more to the total uptake that neuronal EAAT2 (Fig. 7d). This was indeed the case, and upon reconstituting the transporters, the transport activity then matched the Western blots [204].

These experiments were repeated with tissue from other brain regions, and it was concluded that there is EAAT2 in nerve terminals in most parts of the brain, but it was also concluded that the Syn1-cre line used (RRID:IMSR_JAX:003966; [207]) did not delete the floxed-EAAT2 in all neurons implying that the contribution of neuronal EAAT2 is somewhat underestimated [9].

However, a differential resealing probability can not explain the high d-Asp uptake rate into axon-terminals in hippocampal tissue slices because astrocytes in such slice preparations are able to maintain their electrochemical transmembrane gradients. Thus, there have to be additional factors and exchange of external substrates with internal substrates in a 1:1 relationship might be one of them.

An external substrate can be taken up by cells in a tissue slice by two different mechanisms [55]: net uptake (external substrate and Na^+^ are taken up while internal K^+^ is released) and exchange (uptake of one external substrate molecules results in the release of one internal molecule so that the total numbers of substrate molecules on each side of the membrane remain unchanged). Exchange of d-Asp with internal l-Glu is referred to as heteroexchange, while exchange of l-Glu with l-Glu is homoexchange.

If only net uptake took place, then astroglia would be expected to accumulate faster than terminals due to a higher number of transporter molecules. Exchange on the other hand might be expected to affect terminals and astrocytes differently. Because terminals have high levels of internal l-Glu, it follows that added external d-Asp is likely to exchange with internal l-Glu because the number of internal l-Glu molecules inside is far higher than the number of d-Asp molecules. Thus, heteroexchange with terminals will cause l-Glu release while d-Asp is trapped inside. In contrast, because of lower levels of internal l-Glu in astrocytes, exchange may not lead to efficient accumulation of d-Asp because d-Asp may exchange with a d-Asp molecule already taken up. Further, any l-Glu released from terminals due to heteroexchange, may bind to glial EAAT2 transporters and exchange with accumulated d-Asp thereby causing net d-Asp loss from astrocytes thereby causing in underestimation of the rate of net uptake [194].

### The Relative Rates of Net Uptake and Heteroexchange

**Fig. 8:**
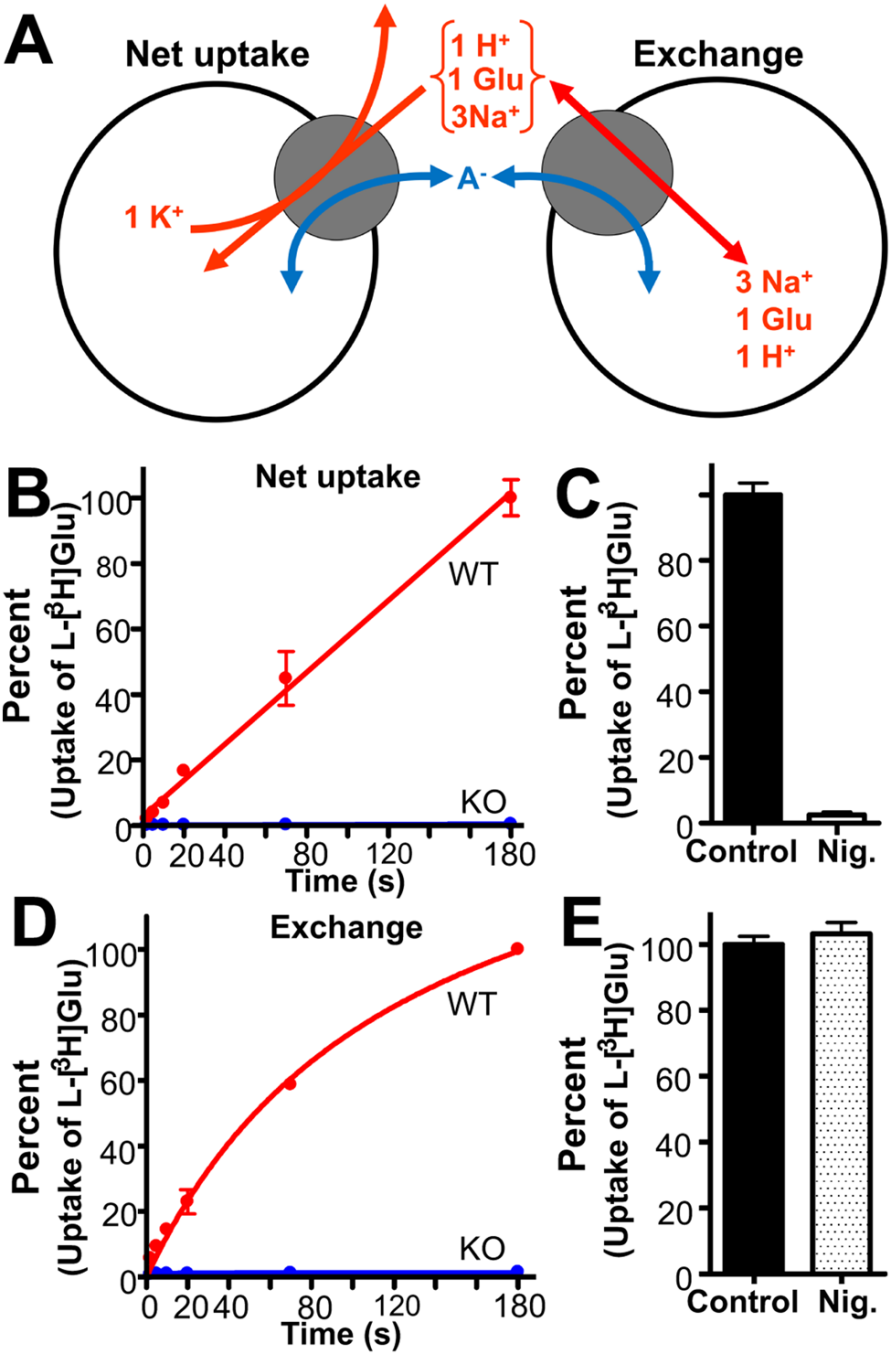
**a** Two modes of glutamate translocation [55]. Large open circles represent liposomes, and smaller filled gray circles represent transporter molecules. Stoichiometric transport is indicated with red arrows and uncoupled anion (A^−^) fluxes by blue arrows. Net uptake is a process in which external substrate is taken up in a manner dependent on internal K^+^. This leads to net removal of substrate from the extracellular fluid. In contrast, (hetero)exchange is a process in which internal unlabeled substrate is exchanged with external labeled substrate in a 1:1 relationship. The latter process does not alter the number of substrate molecules on each side of the membrane but allows molecules to switch location. This process requires Na^+^, but not K^+^, and occurs in the absence of transmembrane gradients. Consequently, dissipation of transmembrane ion gradients with the ionophore nigericin (Nig) abolishes net uptake (**c**), but does not have much effect on exchange (**e**). In the absence of K^+^, the transporters are locked in exchange mode. An important consequence is the following: when transportable uptake inhibitors are added to cell cultures, the inhibitors induce glutamate release from the cells (e.g. [25, 55]). Panels **b** and **d**: The glutamate uptake activity measured with the reconstituted system is mostly due to EAAT2 when forebrain tissue is used as the source of transporter proteins. **b** Potassium-loaded proteoliposomes prepared from wild-type (WT) and EAAT2 knockout mice (KO [199]) were diluted into a large volume of a sodium-containing medium with radiolabeled glutamate to measure net uptake. Internal medium: 135 mM KPi (pH7.4) with 1 % (v/v) glycerol. External medium: 135 mM NaPi (pH7.4), 1 % (v/v) glycerol, 3 µM valinomycin, 2 µM Na-glutamate, and 1.4 µCi l-[^3^ H]glutamate. Liposomes prepared from the EAAT2-deficient animals (KO) had little uptake activities compared with those prepared from WT littermates. **d** Sodium- and glutamate-loaded proteoliposomes, prepared from WT and KO mice, were diluted into a large volume of a sodium containing medium with radiolabeled glutamate to measure heteroexchange. Internal medium: 120 mM NaPi (pH7.4), 20 mM Na-glutamate, and 1 % (v/v) glycerol. External medium: 135 mM NaPi (pH7.4), 1 % (v/v) glycerol, 2 µM Na-glutamate, and 1.4 µCi l-[^3^ H]glutamate. **c** Addition of nigericin (Nig.) to potassium-loaded proteoliposomes abolishes the uptake activities. **e** Glutamate uptake activities by proteoliposomes loaded with sodium and glutamate are insensitive to nigericin. (Copyright: The figure was reproduced from Fig. 1 in Zhou et al. [19])

**Fig. 9:**
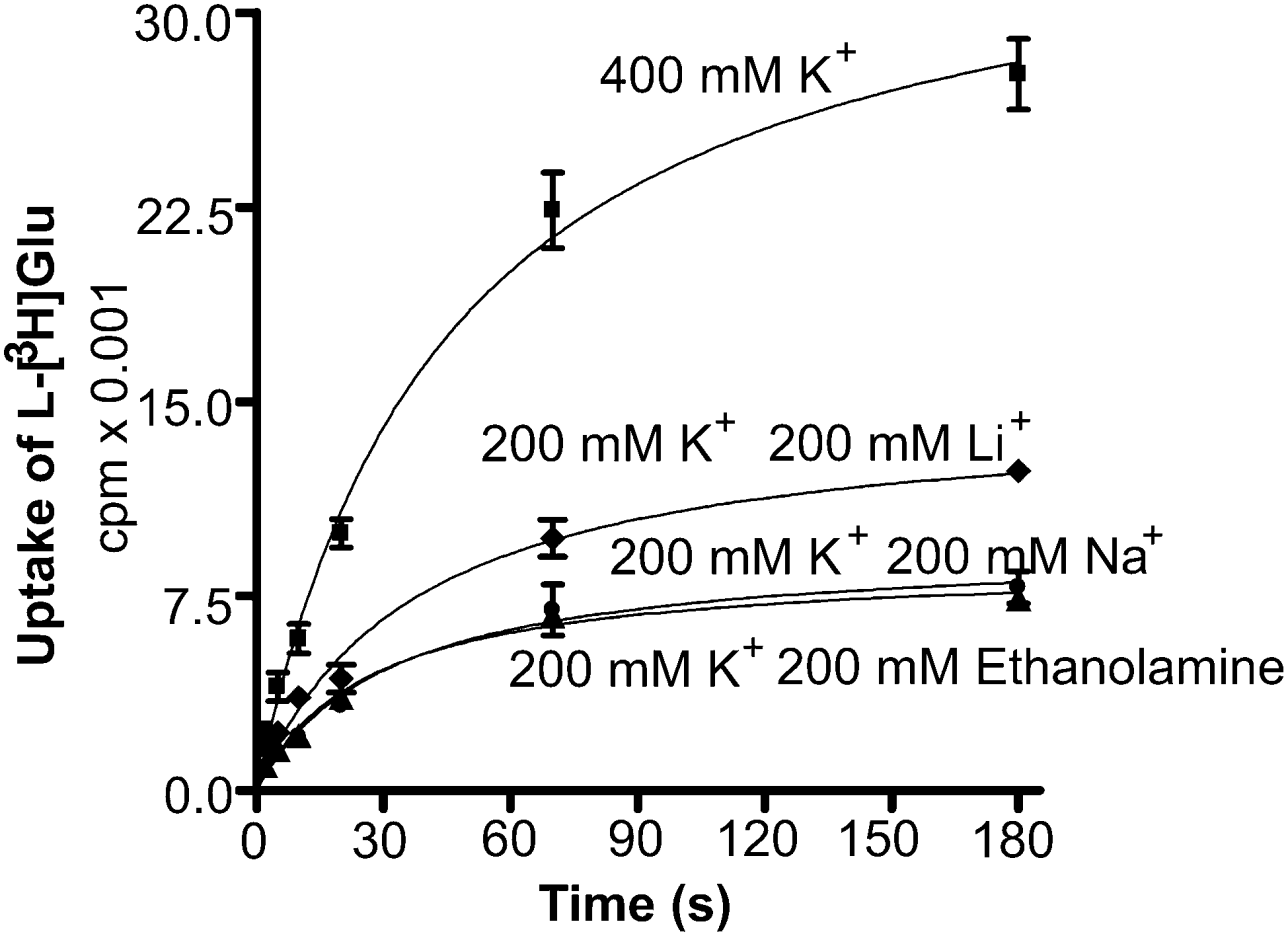
The effect of intraliposomal potassium on the rate of net uptake. Proteoliposomes were loaded with various internal media: 400 mM K^+^ (400 mM potassium ions, 250 mM HEPES, 100 mM citric acid, and 1% v/v glycerol), 200 mM K^+^, 200 mM Li^+^ (200 mM potassium ions, 200 mM lithium ions, 250 mM HEPES, 100 mM citric acid, and 1% v/v glycerol), 200 mM K^+^, 200 mM Na+ (200 mM potassium ions, 200 mM sodium ions, 250 mM HEPES, 100 mM citric acid, and 1% v/v glycerol); 200 mM K^+^, 200 mM ethanolamine (200 mM potassium ions, 200 mM ethanolamine, 250 mM HEPES, 100 mM citric acid, and 1% v/v glycerol). They were added into external medium containing 400 mM sodium ions, 250 mM HEPES, 100 mM citric acid, 1% v/v glycerol, 2 µM Na-glutamate, 50 nM l-[^3^ H]glutamate, and 3 µM valinomycin. The data represent average ± SEM of one experiment with triplicates. (Copyright: The figure was reproduced from Fig. 7A1 in Zhou et al. [19])

**Fig. 10:**
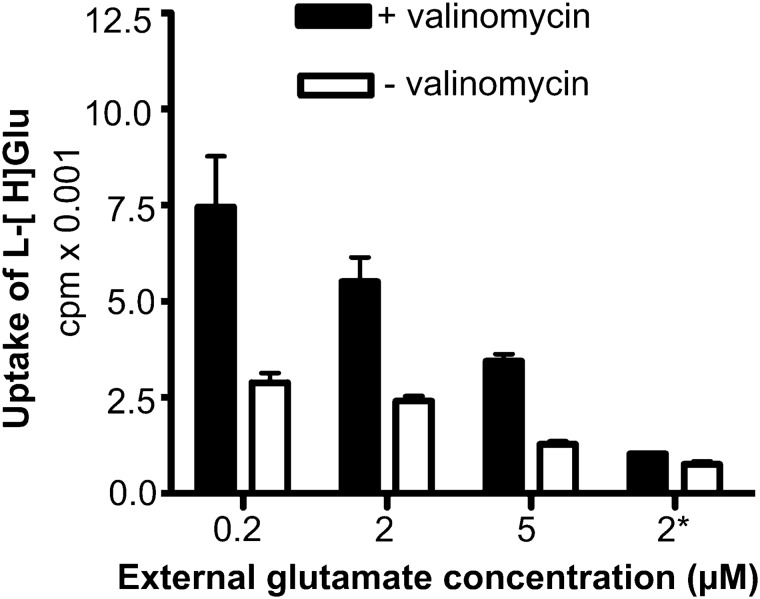
Stimulation of net uptake by the negative membrane potential created by the addition of the potassium ionophore valinomycin. Proteoliposomes were loaded with 20 mM K-gluconate, 15 mM KPi (pH7.4), 145 mM KCl and 1% (v/v) glycerol). The reactions were started by the addition of proteoliposomes into reaction buffers with varying concentrations of external glutamate (0.2, 2 or 5 µM unlabeled Na-glutamate, 50 nM l-[^3^ H]glutamate, 20 mM Na-gluconate, 15 mM NaPi (pH7.4), 145 mM NaCl, and 1% (v/v) glycerol. The addition of 3 µM valinomycin was indicated as a solid bar. The incubation time was 5 s. 3 µM nigericin was added as negative control (*). The figure represents average ± SEM of one representative experiment (n = 3 replicates). (Copyright: The figure was reproduced from Fig. 2B in Zhou et al. [19])

**Fig. 11:**
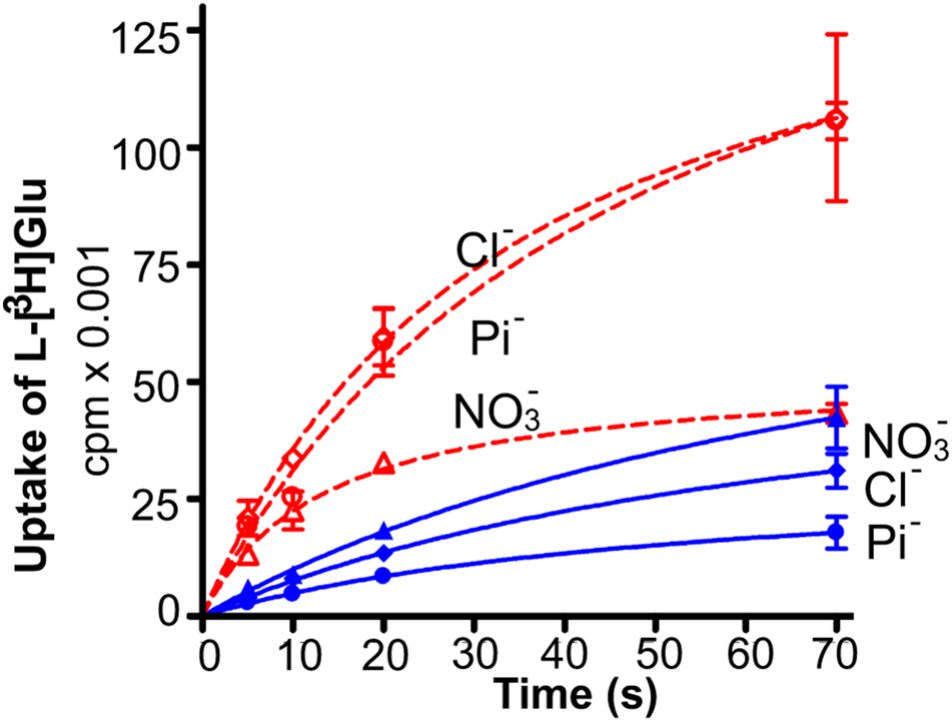
The effect of membrane potential built by anions on the rate of net uptake. Proteoliposomes were loaded with various internal media: NO_3_^−^ (100 mM KNO_3_, 20 mM KPi pH 7.4, and 1% v/v glycerol), Cl^−^ (100 mM KCl, 20 mM KPi pH 7.4, and 1% v/v glycerol), Pi- (100 mM KPi pH7.4, and 1% v/v glycerol). Then they were added into related external media containing 2 µM l-[^3^ H]glutamate and sodium without valinomycin (blue solid line) or with 3 µM valinomycin (dashed red line). (Copyright: The figure was reproduced from Fig. 7a in Zhou et al. [19])

**Fig. 12:**
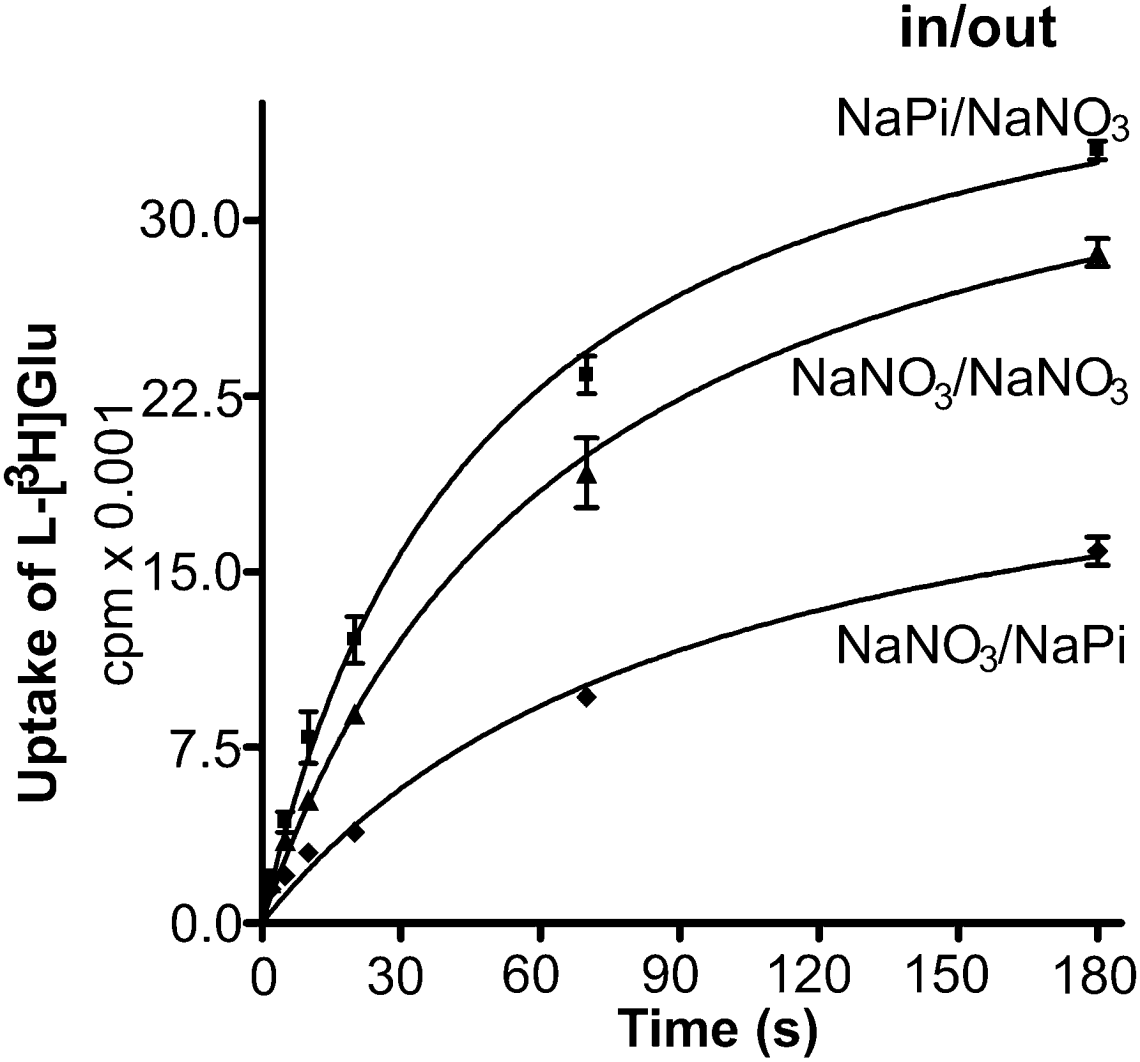
Electroneutral glutamate exchange is voltage dependent. Proteoliposomes were loaded (indicated as “in”) with either: NaPi (120 mM NaPi pH7.4, 20 mM Na-glutamate, and 1% v/v glycerol) or NaNO_3_ (100 mM NaNO_3_, 40 mM NaPi pH 7.4, 20 mM Na-glutamate and 1% v/v glycerol), and then diluted into external media containing 2 µM l-[^3^ H]glutamate and either NaNO_3_ (100 mM NaNO_3_, 40 mM NaPi pH 7.4, 20 mM Na-gluconate and 1% v/v glycerol), or NaPi (120 mM NaPi pH7.4, 20 mM Na-gluconate, and 1% v/v glycerol). (Copyright: The figure was reproduced from Fig. 5C in Zhou et al. [19])

**Fig. 13:**
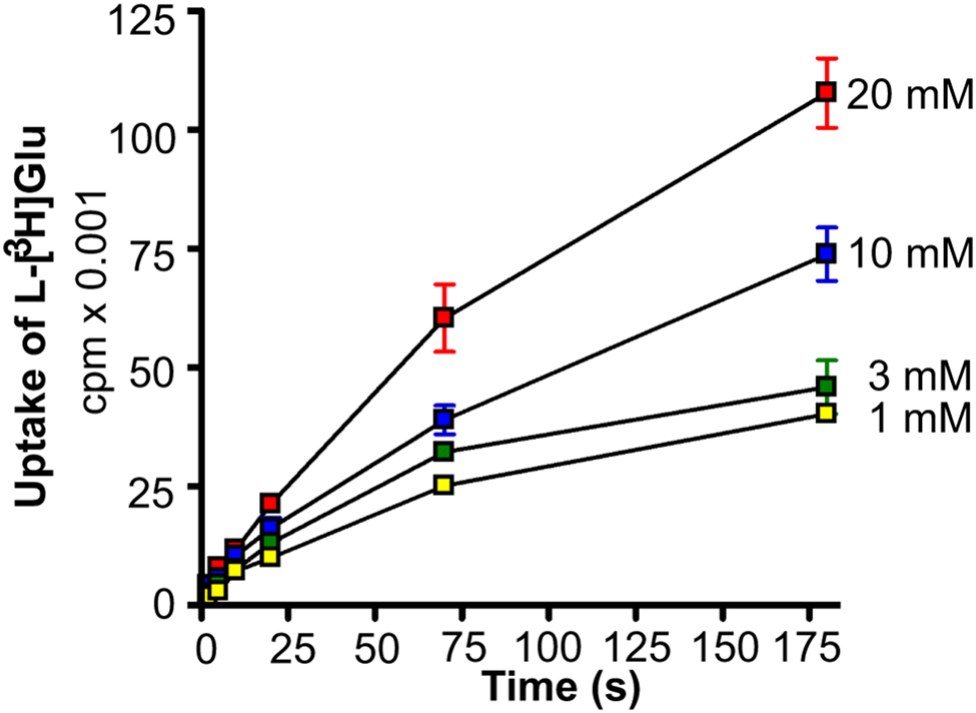
The effect of intraliposomal glutamate on the exchange rate. Proteoliposomes were loaded with internal media consisting of 15 mM KPi (pH 7.4), 145 mM KCl and the indicated concentrations of unlabeled l-Glu (1 20 mM). The liposomes (20 µl) were diluted into 1500 µl of external medium consisting of 15 mM NaPi (pH 7.4), 145 mM NaCl, 2 µM labeled L[^3^H]glutamate. Sodium gluconate was added to balance the osmolarity of the internal glutamate. The uptake reaction was stopped by dilution and filtration after the time indicated (0–180 s). Note that external substrate (L[^3^ H]glutamate, 2 µM) enters faster into liposomes if the liposomes already contain high substrate concentrations. The figure represents unpublished data from the study of the relative rates of net uptake and heteroexchange [19]. Copyright: Neurotransporter.org; reproduced with permission

Another parameter is how fast exchange is relative to net uptake. To address this issue, a reconstituted system was used to compare the relative rates of the two processes (Fig. 8, also see: [19]). This revealed a complex picture of how the EAAT2 transporter protein functions. Net uptake was in this study found to be highly dependent on internal potassium (Fig. 9), to be sensitive to changes in the membrane potential (Fig. 10), and to be stimulated by external permeable anions in agreement with the existence of an uncoupled anion conductance (Fig. 11). By utilizing the latter, we also demonstrated that the rate of heteroexchange depends on the nature of the internal anion (presumably the membrane potential; Fig. 12) as well as intracellular substrate (glutamate) concentrations (Fig. 13). When considering all these parameters, contrary to expectations, rates of uptake and heteroexchange are similar at low glutamate concentration and only a factor of two different at high glutamate concentration [19]. Our simulations suggested that the reason why exchange is not much faster than net uptake under these conditions is that the transporter in exchange mode populates with similar probability at a number of states. Because the rates between these states are all very similar, we suggest that the transporter undergoes a random walk similar to the “Drunkard’s walk” (equal probability to step forward as backward). This may significantly prolong the time it takes to complete an exchange of a glutamate molecule because, for each transition, the transporter hops back and forth so much that it adds up to a substantial time on average. Therefore, our model suggests that glutamate exchange is slow because of the large number of relatively fast steps (random forward and backward steps), whereas net uptake is similarly slow because of one slow rate-limiting K^+^-relocation step (for a full discussion, see: [19].

### Transportable Uptake Inhibitors are not Uptake Blockers

**Fig. 14:**
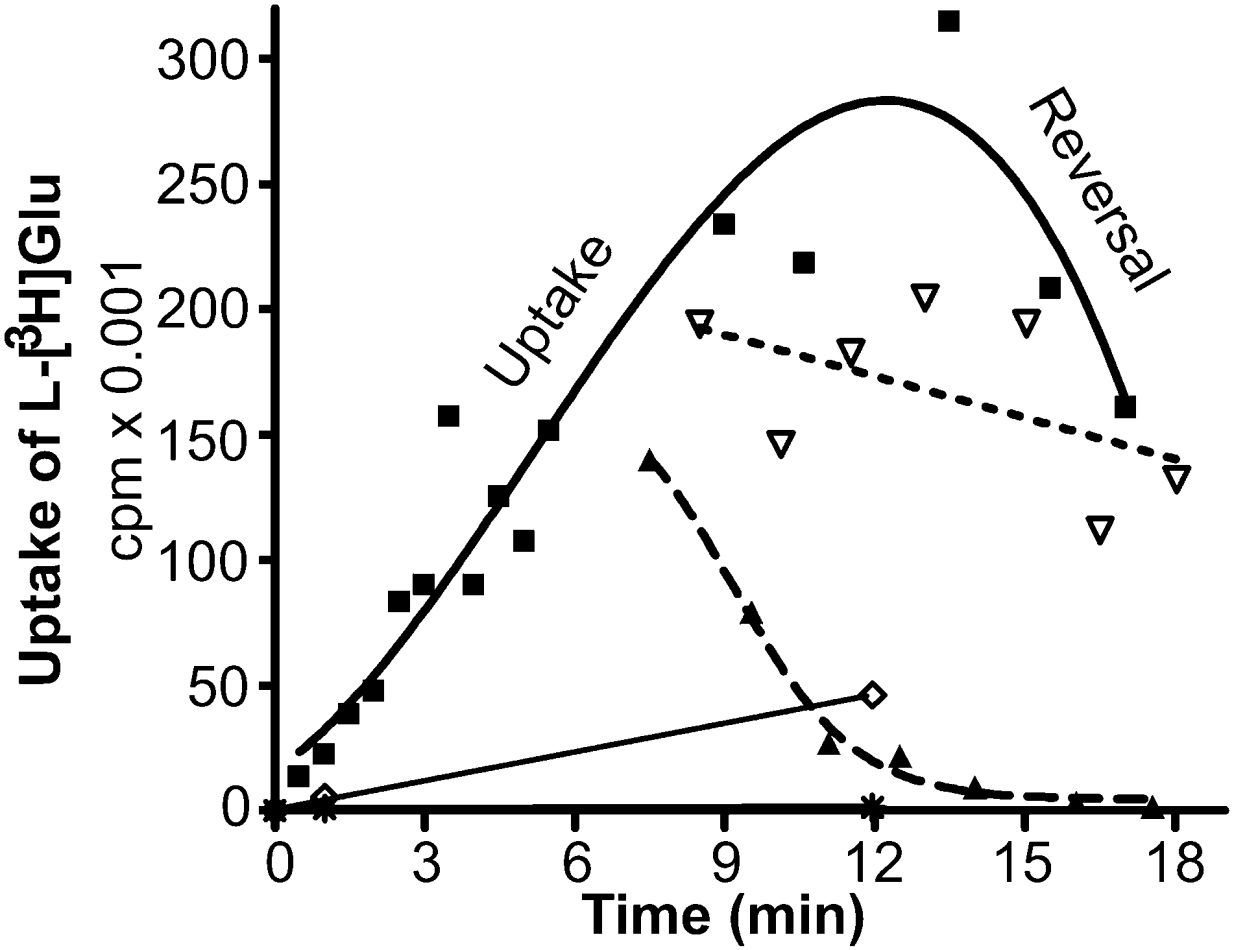
Bell-shaped time course due to uptake followed by reversed uptake. Liposomes are loaded with the standard K^+^-containing internal medium (0.12 M potassium phosphate with 1 ml/dl glycerol) and the uptake reaction is started by diluting the liposomes in Na^+^-containing external medium (0.15 M NaCl with 1 ml/dl glycerol, 3 µM valinomycin) with 50 nM l-[^3^H]glutamate). Glutamate accumulates inside the liposomes (solid squares) until the ion gradients are dissipated. Then glutamate leaks out presumably to reversal of the transporters giving rise to the bell-shaped time course. Addition of nigericin abolishes uptake when added at start (asterisks) and induces reversal when added later (solid triangles). Dihydrokainate inhibits uptake (open squares) but is also able to block reversal because it is a non-transportable substrate as illustrated by the addition of both nigericin and dihydrokainate (open triangles). Data from the same material as in [224]). Copyright: The figure was reproduced from Fig. 4E in Zhou et al. [19]

When a transportable compound is used to inhibit uptake by competitive inhibition, the result is not only inhibition of removal of external l-Glu, but also release of internal l-Glu due to heteroexchange [25, 212, 213]. In contrast, non-transportable inhibitors (Fig. 14) block both net uptake and heteroexchange [55].

### Contribution of the Reconstitution Method to Validate Transporter Structural 3D Models

As already explained, the pioneer work of Kanner using reconstitution assays permitted the purification and cloning of GAT1 (Slc6a1). Although important in itself, this also provided sequence probes for the cloning of other SLC6 members. Further, the purification and cloning of EAAT2 (GLT-1; Slc1a2) allowing the study of multiple functional properties (see above). In addition, Kanner´s reconstitution method was also extremely useful to validate the features of the 3D structural models for the transporters when crystals were available [41, 107, 108]. One of the most important contributions was the identification of the chloride binding site of the SLC6 transporters. By introducing a negatively charged substitution (mimicking the Cl^−^ ion) in the bacterial LeuTAa analog, it was shown that the chloride-independent transport by the mutant acquired chloride dependence in proteoliposomes. Furthermore, lowering the internal proteoliposome pH in reconstituted transporters, which was expected to neutralize the introduced negative charge, increased the rate of transport of the mutant, but not of the wild-type, by an order of magnitude. These experiments, performed on proteoliposomes, provided a fundamental piece of evidence for the location of the chloride site in GAT1 [214] at an equivalent position as was located in SERT [215] and GlyTs [216].

In subsequent work dealing with structure-function of the transporters, the reconstitution assay was successfully used as a method to differentiate transporter mutations which impair transport activity from those impairing transporter intracellular trafficking. By reconstituting membrane proteins from cells heterologously expressing transporter mutants, cryptic transport activities of transporter proteins retained in intracellular membranes could be detected [217–219]. These studies permitted, for example, the identification of key residues included in the substrate-binding site of GAT-1 conserved in the whole SLC6 family [68].

Reconstitution was also the method of choice for studying the properties of the intracellular gate of SLC6 transporters. Mutation of two conserved residues in the cytoplasmic amino-terminal tail of GAT-1 yielded transporters defective in net GABA transport but capable of sodium-dependent exchange of [^3^H]-GABA with unlabeled GABA, a property that could only be measured in proteoliposomes. This work identified a consensus sequence that influences the reorientation step of the SLC6 transporters [220, 221].

In the glutamate GLT-1 transporter, the proximity of two oppositely oriented reentrant loops flanking the substrate binding site (HP1 and HP2) was deduced by paired cysteine mutagenesis by Kanner´s group after realizing the double cysteine mutant was retained in intracellular compartments using cell reconstitution [222]. The conclusions of this study were later confirmed when data from the first glutamate transporter crystal were available [53] and later when the human EAAT1 was crystallized to a higher resolution [41]. Many other achievements were reached by Kanner´s group regarding GABA and glutamate transporters structure–function using reconstitution, but also electrophysiological techniques. One can say without fear of exaggeration that Kanner has settled the basis for understanding the coupling between ions and substrate in the neurotransmitter transporters [223].

## Concluding Remarks

Kanner’s early work on methods to study transporter proteins had a huge scientific impact as it led to the isolation and cloning of the first neurotransmitter transporter, GAT1 (Slc6a1 [70]) and to the isolation [13] and cloning of the major brain glutamate transporter, EAAT2 (Slc1a2 [50]) as well as the isolation of GlyT2 [21]. Further, these methods also enabled the early description of these transporters’ nature and their further characterization as illustrated in Fig. 14. The understanding obtained was later confirmed by structural and electrophysiological techniques. The latter techniques are superior when it comes to temporal resolution, but depend on detection of charge transfer and are thereby unable to study electroneutral processes in contrast to the reconstitution assay described here. The reconstitution assay also has advantages when it comes to screening of a large number of samples. Thus, Kanner’s early contribution has been very important. The scope of this review is not to cover Kanner’s later work which also has had a huge impact.

## Data Availability

The manuscript has no associated data.

## Abbreviations

CAS: Chemical abstracts service registry number
CHAPS: 3-[(3-Cholamidopropyl)dimethylammonio]-1-propanesulfonate
CMC: Critical micellar concentration
DTT: Dithiothreitol
EAAT: Excitatory amino acid (glutamate) transporter
EDTA: Ethylenediaminetetraacetic acid
KPi: Potassium phosphate buffer
NaPi: Sodium phosphate buffer
PMSF: Phenylmethanesulfonyl fluoride
